# Synthesis and computational evaluation of the antioxidant activity of pyrrolo[2,3-*b*]quinoxaline derivatives†

**DOI:** 10.1039/d4ra03108c

**Published:** 2024-08-05

**Authors:** Nguyen Tran Nguyen, Vo Viet Dai, Adam Mechler, Luc Van Meervelt, Nguyen Thi Hoa, Quan V. Vo

**Affiliations:** a The University of Danang-University of Science and Education Danang 550000 Vietnam ntnguyen@ued.udn.vn; b Department of Biochemistry and Chemistry, La Trobe University Victoria 3086 Australia; c Biomolecular Architecture, Department of Chemistry, KU Leuven Celestijnenlaan 200F B-3001 Leuven Belgium; d The University of Danang – University of Technology and Education Danang 550000 Vietnam vvquan@ute.udn.vn

## Abstract

Pyrrolo[2,3-*b*]quinoxaline derivatives are known to possess antioxidant, anticancer, and antibacterial properties. Here we report the successful synthesis of five derivatives of 3-hydroxy-3-pyrroline-2-one through substitution. The 2,2-diphenyl-1-picrylhydrazyl (DPPH) assay was employed to evaluate the antioxidant activity of the compounds. Out of these, ethyl 1,2-diphenyl-1*H*-pyrrolo[2,3-*b*]quinoxaline-3-carboxylate (3a) demonstrated the greatest potential as a radical scavenger. Thermodynamic and kinetic calculations of the radical scavenging activity indicated that 3a exhibited HO˙ radical scavenging activity with the overall rate constant of 8.56 × 10^8^ M^−1^ s^−1^ in pentyl ethanoate; however, it was incapable of scavenging hydroperoxyl radicals in nonpolar media. In non-polar environments, the hydroxyl radical scavenging capability of 3a is fairly similar to that of reference antioxidants such as Trolox, melatonin, indole-3-carbinol, and gallic acid. Hence, in the physiological lipid environment, 3a holds promise as a scavenger of HO˙ radicals.

## Abstract 2

The antioxidant activity of five derivatives of 3-hydroxy-3-pyrroline-2-one was effectively assessed after synthesis.

## Graphical abstract

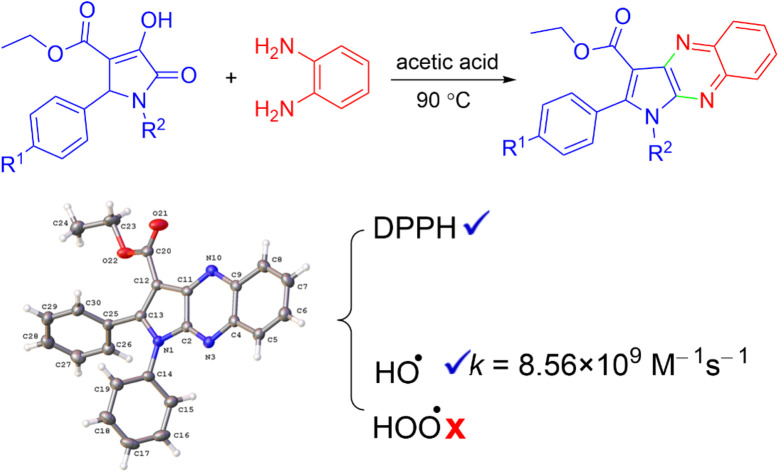

## 1. Introduction

**Fig. 1:**
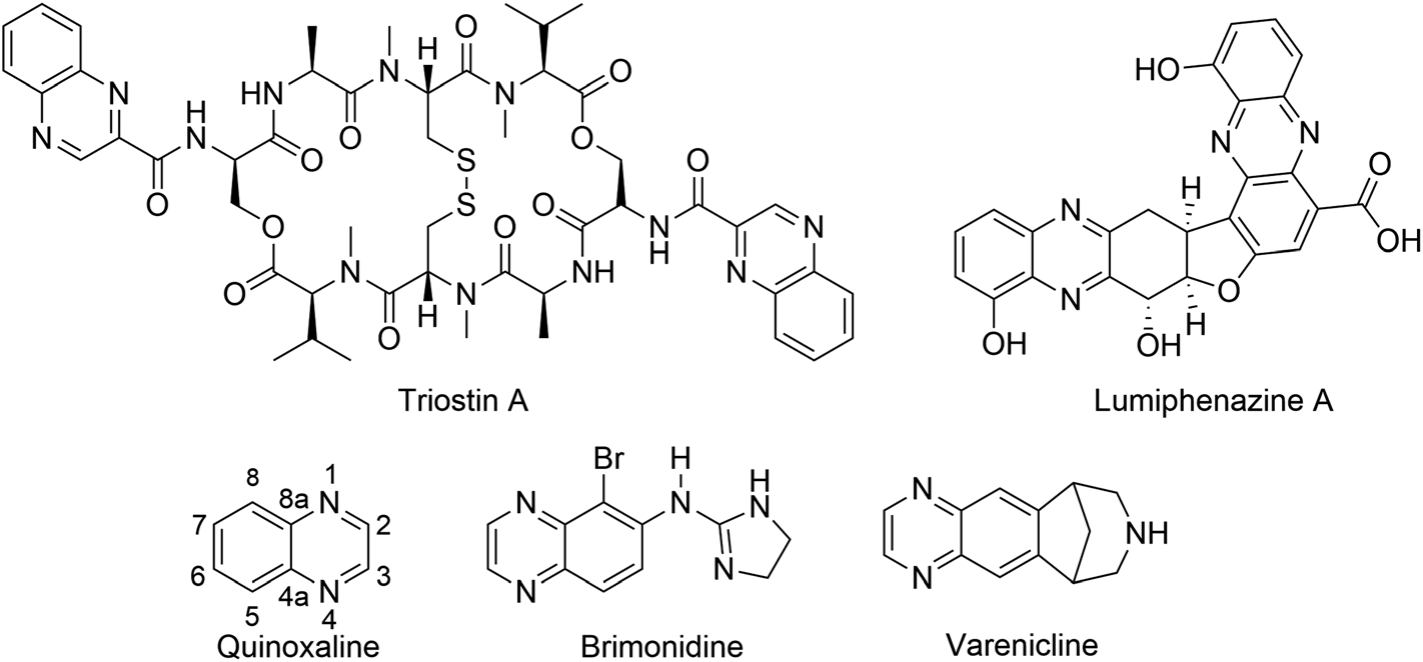
Representative examples of natural products and commercially available drugs containing quinoxaline moiety.

The quinoxaline nucleus exists in numerous bioactive natural products. For instance, *Lumiphenazine A* isolated from *Streptomyces* sp. IFM 11204 showed biological activity against human gastric adenocarcinoma cells.^1^ Triostin A isolated from *Streptomyces aureus* S-2-210 exhibited antitumor and antimicrobial activities.^2,3^ In addition to natural products, several quinoxaline-containing synthetic drugs have been discovered.^4–7^ Bromonidine, for instance, is a commercially available α-2 adrenergic agonist which has been used to treat glaucoma and ocular hypertension, as well as facial erythema in rosacea.^6^ Varenicline is a quinoxaline-based α4β2 nicotinic receptor partial agonist drug available in the market and is used for smoking cessation (Fig. 1).^5^

It should be noted that most pyrrolo[2,3-*b*]quinoxalines have been synthesized from halogen-containing quinoxaline derivatives with palladium catalysts. For instance, reactions of 2-alkynyl-3-trifluoroacetamido quinoxalines with aryl and vinyl halides in the presence of Pd(PPh_3_)_4_ and K_2_CO_3_,^8^ Sonogashira coupling reaction of *N*-alkyl-3-chloroquinoxaline-2-amines with propargyl bromide in the presence of PdCl_2_(PPh_3_)_2_ and CuI,^9^ and Pd/C-catalyzed reactions of dichloroquinoxaline with hydrazine, phenylacetylene and aldehydes,^10^ Recently, bismuth salts Bi(OTf)_3_ were used as the catalyst for the condensation of 1*H*-pyrrole-2,3-diones and *o*-phenylenediamine to yield pyrrolo[2,3-*b*]quinoxalines.^11^ Biological and pharmacological activities of synthetic pyrrolo[2,3-*b*]quinoxalines were also reported identifying these compounds as anticancer agents,^12,13^ antibiotics,^14,15^ and antioxidants.^16^

Polysubstituted 3-hydroxy-3-pyrroline-2-one has recently appeared as an effective 1,2-diketone-containing building block for the synthesis of pyrrolo[2,3-*b*]quinoxalines.^16^ These 2-oxopyrrole-containing compounds also possess a wide array of biological activities on their own, *e.g.*, anticancer,^17,18^ anti-bacterial,^19,20^ anti-HIV,^21^ anti-inflammatory,^22^ and antioxidant^23,24^ activities. Therefore, the synthesis of quinoxaline derivatives starting from polysubstituted 3-hydroxy-3-pyrroline-2-ones can yield pyrrolo[2,3-*b*]quinoxalines with potential biological activities. In this manuscript, we report the synthesis of pyrrolo[2,3-*b*]quinoxalines *via* the condensation of 1,5-disubstituted-4-ethoxycarbonyl-3-hydroxy-3-pyrroline-2-one with *o*-phenylenediamine. The 1,1-diphenyl-1-picrylhydrazyl (DPPH) free radical scavenging capacity of pyrrolo[2,3-*b*]quinoxalines is discussed. Furthermore, the interaction of the best-performing pyrrolo[2,3-*b*]quinoxaline with HO˙ and HOO˙ radicals is evaluated using well-established model chemistry based on the quantum mechanics-based test for the overall free radical scavenging activity (QM-ORSA) protocol.

## 2. Experimental and computational methods

### 2.1. Experimental

#### 2.1.1. Chemicals and experimental methods

All chemicals were purchased from Merck, Acros, or Sigma-Aldrich without further purification. 70–230 mesh silica 60 (E. M. Merck) was used as the stationary phase for column chromatography. NMR spectra were acquired on Bruker Avance II+ 600 MHz instruments. The chemical shifts (*δ*) were reported in parts per million (ppm) relative to tetramethylsilane (TMS) or the internal (NMR) solvent signals. High-resolution mass spectra (ESI-quadrupole) of compounds 3a, 3b and 3e were measured on a quadrupole orthogonal acceleration time-of-flight mass spectrometer (Synapt G2 HDMS, Waters, Milford, MA). In addition, high-resolution mass spectra (ESI-TOF MS/MS) of compounds 3a′, 3c and 3d were also recorded on a SCIEX X500 QTOF with an electrospray ionization source in a positive ion mode. Büchi Melting Point B-545 apparatus was used to determine the melting points of all products.

#### 2.1.2. General procedure for the synthesis of pyrrolo[2,3-*b*]quinoxalines 3a–e

1,5-Disubstituted-4-ethoxycarbonyl-3-hydroxy-3-pyrroline-2-one^24^ (0.155 mmol, 1 equiv.) and *o*-phenylenediamine (0.464 mmol, 3 equiv.) were added to a screw-capped reaction tube equipped with a magnetic stirring bar. Subsequently, glacial acetic acid (0.5 mL) was added and the mixture was stirred vigorously at 90 °C for 2–8 hour. The consumption of reactants and the formation of the product was followed by TLC (hexane/ethyl acetate). Distilled water was then added to the tube and the mixture was stirred at room temperature for 15 minutes. The precipitate was purified by column chromatography (silica gel, eluent hexane/ethyl acetate). In some cases, recrystallization was used for further purification after doing column chromatography.

#### 2.1.3. General procedure for the synthesis of pyrrolo[2,3-*b*]quinoxaline 3a′

1,5-Diphenyl-4-ethoxycarbonyl-3-hydroxy-3-pyrroline-2-one^24^ (0.155 mmol, 1 equiv.), *o*-phenylenediamine (0.464 mmol, 3 equiv.) and citric acid (0.31 mmol, 2 equiv.) were added to a screw-capped reaction tube equipped with a magnetic stirring bar. Subsequently, absolute ethanol (1 mL) was added and the reaction mixture was stirred vigorously at 80 °C for 3 hours. Then, distilled water was added to the tube and the mixture was stirred at room temperature for 15 minutes. The precipitate was purified by column chromatography (silica gel, dichloromethane).

#### 2.1.4. Spectra data of products

##### (a) Ethyl 1,2-diphenyl-1*H*-pyrrolo[2,3-*b*]quinoxaline-3-carboxylate (3a)

^1^H NMR (600 MHz, CDCl_3_) *δ* 8.41 (dd, ^3^*J*(H,H) = 8.28 Hz, ^4^*J*(H,H) = 1.32 Hz, 1H; Ar–H), 8.05 (dd, ^3^*J*(H,H) = 8.27 Hz, ^4^*J*(H,H) = 1.37 Hz, 1H; Ar–H), 7.71 (m, 2H; Ar–H), 7.38 (m, 6H; Ar–H), 7.27 (m, 2H; Ar–H), 4.32 (q, ^3^*J*(H,H) = 7.14 Hz, 2H; OCH_2_), 1.19 ppm (t, ^3^*J*(H,H) = 7.14 Hz, 3H; CH_3_). ^13^C NMR (150 MHz, CDCl_3_) 163.34, 155.14, 142.87, 142.27, 140.63, 139.84, 134.93, 130.70, 130.15, 130.07, 129.70, 129.21, 128.79, 128.72, 128.53, 128.45, 127.90, 127.75, 105.31, 60.54, 14.19 ppm. HRMS (ESI-quadrupole) *m*/*z* [M + H]^+^ calcd for C_25_H_19_N_3_O_2_: 394.1555; found: 394.1540.

##### (b) Ethyl 1,2-diphenyl-2,4-dihydro-1*H*-pyrrolo[2,3-*b*]quinoxaline-3-carboxylate (3a′)

^1^H NMR (600 MHz, CDCl_3_) *δ* 8.96 (s_br_, 1H; NH), 7.77–6.90 (14H; Ar–H), 6.25 (s, 1H), 4.12 (q, ^3^*J*(H,H) = 7.05 Hz, 2H; O–CH_2_), 1.18 ppm (t, ^3^*J*(H,H) = 7.10 Hz, 3H; CH_3_). ^13^C NMR (150 MHz, CDCl_3_) 155.75, 139.30, 138.61, 135.95, 128.85, 128.36, 127.90, 127.42, 127.39, 126.60, 124.95, 124.26, 123.71, 121.54, 114.60, 96.61, 67.43, 59.86, 14.39 ppm. HRMS (ESI-TOF MS/MS) *m*/*z* [M + H]^+^ calcd for C_25_H_21_N_3_O_2_: 396.1712; found: 396.1682.

##### (c) Ethyl 1-(3-nitrophenyl)-2-(*p*-tolyl)-1*H*-pyrrolo[2,3-*b*]quinoxaline-3-carboxylate (3b)

^1^H NMR (600 MHz, CDCl_3_) *δ* 8.41 (dd, ^3^*J*(H,H) = 8.22 Hz, ^4^*J*(H,H) = 1.60 Hz, 1H; Ar–H), 8.22 (m, 2H; Ar–H), 8.03 (dd, ^3^*J*(H,H) = 8.23 Hz, ^4^*J*(H,H) = 1.65 Hz, 1H; Ar–H), 7.74 (m, 2H; Ar–H), 7.59 (m, 2H; Ar–H), 7.27 (d, ^3^*J*(H,H) = 8.12 Hz, 2H; Ar–H), 7.18 (d, ^3^*J*(H,H) = 7.86 Hz, 2H; Ar–H), 4.37 (q, ^3^*J*(H,H) = 7.13 Hz, 2H; OCH_2_), 2.37 (s, 3H; CH_3_), 1.25 ppm (t, ^3^*J*(H,H) = 7.10 Hz, 3H; CH_3_). ^13^C NMR (150 MHz, CDCl_3_) 163.14, 154.44, 148.58, 142.53, 142.46, 140.62, 140.60, 139.62, 136.25, 134.45, 130.66, 130.17, 129.94, 129.10, 128.98, 128.59, 128.13, 126.29, 123.85, 122.96, 106.46, 60.81, 21.65, 14.27 ppm. HRMS (ESI-quadrupole) *m*/*z* [M + H]^+^ calcd for C_26_H_20_N_4_O_4_: 453.1562; found: 453.1555.

##### (d) Ethyl 2-(4-nitrophenyl)-1-phenyl-1*H*-pyrrolo[2,3-*b*]quinoxaline-3-carboxylate (3c)

^1^H NMR (600 MHz, CDCl_3_) *δ* 8.41 (dd, ^3^*J*(H,H) = 8.22 Hz, ^4^*J*(H,H) = 1.27 Hz, 1H; Ar–H), 8.20 (d, ^3^*J*(H,H) = 8.79 Hz, 2H; Ar–H), 8.06 (dd, ^3^*J*(H,H) = 8.16 Hz, ^4^*J*(H,H) = 1.01 Hz, 1H; Ar–H), 7.75 (m, 2H; Ar–H), 7.59 (d, ^3^*J*(H,H) = 8.82 Hz, 2H; Ar–H), 7.42 (m, 3H; Ar–H), 7.26 (m, 2H; Ar–H), 4.36 (q, ^3^*J*(H,H) = 7.12 Hz, 2H; OCH_2_), 1.26 ppm (t, ^3^*J*(H,H) = 7.12 Hz, 3H; CH_3_). ^13^C NMR (150 MHz, CDCl_3_) 162.91, 151.86, 148.37, 142.76, 142.55, 140.11, 139.90, 136.78, 134.36, 131.95, 130.19, 129.63, 129.13, 129.05, 128.78, 128.70, 128.20, 123.05, 106.27, 60.94, 14.31 ppm. HRMS (ESI-TOF MS/MS) *m*/*z* [M + H]^+^ calcd for C_25_H_18_N_4_O_4_: 439.1406; found: 439.1388.

##### (e) Ethyl 1-phenyl-2-(*p*-tolyl)-1*H*-pyrrolo[2,3-*b*]quinoxaline-3-carboxylate (3d)

^1^H NMR (600 MHz, CDCl_3_) *δ* 8.38 (dd, ^3^*J*(H,H) = 8.59 Hz, ^4^*J*(H,H) = 1.56 Hz, 1H; Ar–H), 8.03 (dd, ^3^*J*(H,H) = 8.41 Hz, ^4^*J*(H,H) = 1.30 Hz, 1H; Ar–H), 7.68 (m, 2H; Ar–H), 7.39 (m, 3H; Ar–H), 7.25 (m, 4H; Ar–H), 7.12 (d, ^3^*J*(H,H) = 7.59 Hz, 2H; Ar–H), 4.35 (q, ^3^*J*(H,H) = 7.09 Hz, 2H; OCH_2_), 2.35 (s, 3H; CH_3_), 1.24 ppm (t, ^3^*J*(H,H) = 7.15 Hz, 3H; CH_3_). ^13^C NMR (150 MHz, CDCl_3_) 163.51, 155.49, 142.90, 142.23, 140.73, 139.93, 139.76, 135.06, 130.65, 130.01, 129.20, 128.81, 128.70, 128.63, 128.40, 128.36, 127.67, 126.97, 105.10, 60.55, 21.61, 14.29 ppm. HRMS (ESI-TOF MS/MS) *m*/*z* [M + H]^+^ calcd for C_26_H_21_N_3_O_4_: 408.1712; found: 408.1691.

##### (f) Ethyl 1-benzyl-2-(4-nitrophenyl)-1*H*-pyrrolo[2,3-*b*]quinoxaline-3-carboxylate (3e)

^1^H NMR (600 MHz, CDCl_3_) *δ* 8.43 (m, 1H; Ar–H), 8.28 (d, ^3^*J*(H,H) = 8.67 Hz, 2H; Ar–H), 8.14 (m, 1H; Ar–H), 7.77 (m, 2H; Ar–H), 7.47 (d, ^3^*J*(H,H) = 8.67 Hz, 2H; Ar–H), 7.17 (m, 3H; Ar–H), 6.83 (dd, ^3^*J*(H,H) = 8.26 Hz, ^4^*J*(H,H) = 1.02 Hz, 2H; Ar–H), 5.48 (s, 2H; CH_2_), 4.26 (q, ^3^*J*(H,H) = 7.11 Hz, 2H; OCH_2_), 1.16 ppm (t, ^3^*J*(H,H) = 7.11 Hz, 3H; CH_3_). ^13^C NMR (150 MHz, CDCl_3_) 162.72, 152.89, 148.71, 142.51, 142.09, 139.91, 139.85, 137.09, 136.39, 131.16, 130.27, 129.07, 128.91, 128.60, 128.18, 128.02, 127.11, 123.27, 105.58, 60.63, 46.39, 29.84, 14.22 ppm. HRMS (ESI-quadrupole) *m*/*z* [M + H]^+^ calcd for C_26_H_20_N_4_O_4_: 453.1562; found: 453.1552.

#### 2.1.5. DPPH assay

Radical scavenging properties of pyrrolo[2,3-*b*]quinoxalines were evaluated against 1,1-diphenyl-2-picrylhydrazyl (DPPH) radical.^25,26^ DPPH solution (1 mM) was prepared in methanol and solutions of each pyrrolo[2,3-*b*]quinoxaline were prepared in DMSO at various concentrations (512, 265, 128, 32 and 8 μg mL^−1^). Then, 200 μL DPPH solution was added to 1.28 μL samples at each tested concentration and the free radical scavenging reactions were carried out on a 96-well plate at 37 °C for 30 minutes. The absorbance was measured at 517 nm wavelength by a BioTek Epoch 2 Microplate Spectrophotometer. The percentage of free radical scavenging was calculated as SP (%) = [(OD_0_ − OD_1_)/OD_0_] × 100, where OD_0_ was defined as the final absorbance of the control reaction with quercetin as the reference antioxidant, and OD_1_ stands for the absorbance in the presence of the sample. Each experiment was repeated three times and quercetin was used as the positive control.

### 2.2. Determination of crystal structure of 3a

Single crystals of 3a suitable for X-ray diffraction were obtained by evaporating a solution in dichloromethane/n-hexane mixture at room temperature. X-ray diffraction patterns were collected at 293(2) K on an Agilent SuperNova diffractometer, equipped with an Eos CCD detector, using Mo Kα radiation (*λ* = 0.71073 Å). The images were interpreted and integrated with the CrysAlisPro and the implemented absorption correction was applied.^27^ Using Olex2,^28^ the structure was solved with the ShelXT^29^ structure solution program using intrinsic phasing and refined with the ShelXL^30^ refinement package using full-matrix least-squares minimization on *F*^2^. Non-hydrogen atoms were anisotropically refined and the hydrogen atoms in the riding mode with isotropic temperature factors were fixed at 1.2 times *U*_eq_ of the parent atoms (1.5 for methyl groups). Table S2† gives crystal data and structure refinement details for 3a.

### 2.3. Computational details

All DFT calculations were carried out with the Gaussian 09 suite of programs.^31^ M06-2X functional^32^ and 6-311++G(d,p) basis set were used for all calculations. The M06-2X functional offers one of the most reliable methods to study the thermodynamics and kinetics of radical reactions.^32–37^ The kinetic calculations were performed following the QM-ORSA protocol,^38,39^ following the literature.^36,37,40–43^ This method has been repeatedly benchmarked against experimental data, delivering results with low errors (*k*_calc_/*k*_exp_ ratio = 1–2.9), particularly in lipid medium.^34,38,39^ As a reference all details of the calculations are shown in Table S1, ESI.†

## 3. Results and discussion

### 3.1. Synthesis of pyrrolo[2,3-*b*]quinoxalines

The reaction between 1,5-diphenyl-4-ethoxycarbonyl-3-hydroxy-3-pyrroline-2-one (1a)^24^ and *o*-phenylenediamine (2) was chosen as the model to optimize the reaction conditions, such as the reactant ratio and solvent. Heating 3-pyrroline-2-one derivative 1a (1 equiv.), *o*-phenylenediamine (2) (1 equiv.) and citric acid catalyst (2 equiv.) in 1 mL of absolute ethanol at 80 °C resulted in the formation of pyrrolo[2,3-*b*]quinoxaline 3a with a yield of only 12% (Table 1). However, keeping the equimolar amounts of starting materials 1a (1 equiv., 0.16 mmol) and 2 (1 equiv., 0.16 mmol) in 1 mL of glacial acetic acid solvent at 90 °C brought a dramatic increase in the yield of ethyl 1-phenyl-2-phenyl-1*H*-pyrrolo[2,3-*b*]quinoxaline-3-carboxylate (3a), 50%. Therefore, acetic acid was chosen as the solvent to optimize the concentration of reactants as well as the reactant ratio. The decrease in the concentration of both reactants 1a and 2 to 0.10 M led to a downward trend in the yield of pyrrolo[2,3-*b*]quinoxaline 3a, 40%. In contrast, there was a slight increase in the yield of desired product 3a, 56%, when the concentration of starting materials in acetic acid increased to 0.31 M.

**Table 1:** Optimization of reaction conditions for the synthesis of 3a

Entry	Solvent	Volume (mL)	Ratio 1a : 2 (equiv.)	Concentration 1a : 2 (mmol mL^−1^)	Temperature (°C)	Time (hour)	Yield (%)
1	Ethanol	1.0	1 : 1	0.16 : 0.16	80 °C	5	12
2	AcOH	1.0	1 : 1	0.16 : 0.16	90 °C	5	50
3	AcOH	0.5	1 : 1	0.31 : 0.31	90 °C	5	56
4	AcOH	0.31	1 : 1	0.5 : 0.5	90 °C	5	53
5	AcOH	1.5	1 : 1	0.10 : 0.10	90 °C	5	40
6	AcOH	0.5	1 : 1.5	0.31 : 0.47	90 °C	5	71
7	AcOH	0.5	1 : 2.0	0.31 : 0.62	90 °C	5	78
8	AcOH	0.5	1 : 3.0	0.31 : 0.93	90 °C	5	83
9	AcOH	0.5	1 : 3.0	0.31 : 0.93	110 °C	5	60

**Scheme 1:**
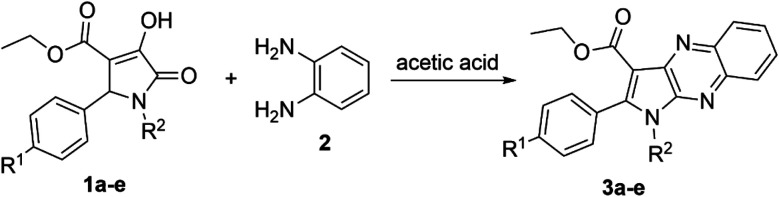
Synthesis of pyrrolo[2,3-*b*]quinoxalines (3a–e).

The product 3a could be obtained in 83% yield when the concentration of aromatic amine 2 in the solvent increased to 0.93 M, while the amount of 3-pyrroline-2-one derivative 1a remained unchanged at 0.31 M. Therefore, the ratio 1 : 3.0 of reactants 1,5-disubstituted-4-ethoxycarbonyl-3-hydroxy-3-pyrroline-2-one and *o*-phenylenediamine, respectively, was used to synthesize other pyrrolo[2,3-*b*]quinoxalines in glacial acetic acid (Scheme 1 and Table 2).

**Table 2:** Synthesis of pyrrolo[2,3-*b*]quinoxalines (3a–e)

Entry	*R* ^1^	*R* ^2^	Time (hour)	Product	Yield (%)
1	H	C_6_H_5_	5	3a	83
2	CH_3_	3-NO_2_C_6_H_4_	8	3b	70.1
3	NO_2_	C_6_H_5_	2	3c	50
4	CH_3_	C_6_H_5_	3	3d	65.2
5	NO_2_	C_6_H_5_CH_2_	3	3e	45

**Scheme 2:**
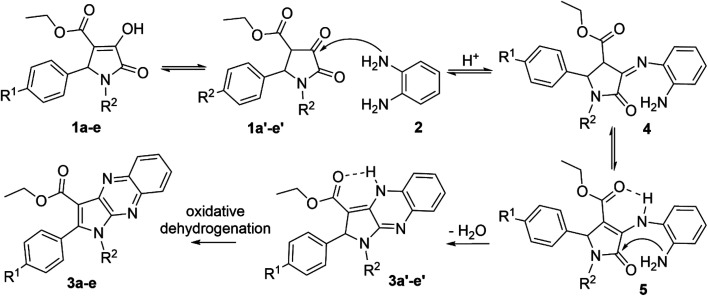
Proposed mechanism for the synthesis of pyrrolo[2,3-*b*]quinoxalines.

The enol tautomer of 1,5-disubstituted-4-ethoxycarbonyl-3-hydroxy-3-pyrroline-2-ones (1a–e) could be converted to the more stable keto form 1a′–e′ in a polar solvent such as ethanol or acetic acid.^44^ The reactions between 1a–e/1a′–e′ and *o*-phenylenediamine (2) are reversible and occur at the 3-position of pyrrolin-2,3-dione heterocyclic ring to yield imine intermediate 4 (ref. 16) Intermediate 4 exists predominantly in the enamine form 5 due to resonance stabilization *via* intramolecular hydrogen bonding^45^ and the intramolecular nucleophilic addition reaction between the amino (NH_2_) and carbonyl group (C=O) in intermediate 5 results in products 3a′–e′. Finally, oxidative dehydrogenation of compounds 3a′–e′ by oxygen molecules in fresh air will result in the formation of pyrrolo[2,3-*b*]quinoxalines 3a–e (Scheme 2).^46^

All new compounds were characterized by NMR and high resolution mass spectrometry (HRMS). In the ^1^H NMR spectrum of ethyl 1,2-diphenyl-2,4-dihydro-1*H*-pyrrolo[2,3-*b*]quinoxaline-3-carboxylate (3a′), there is a characteristic broad and low-intensity peak corresponding to the proton of the secondary amino group (NH) at the chemical shift of 8.97 ppm. Undoubtedly, the intramolecular hydrogen bonding induced the higher chemical shift of the secondary amino proton. Furthermore, the proton at the 2-position of the pyrrole moiety of compound 3a′ appears as a singlet at 6.24 ppm. In contrast, this peak disappears in the ^1^H NMR spectrum of ethyl 1,2-diphenyl-1*H*-pyrrolo[2,3-*b*]quinoxaline-3-carboxylate (3a). Moreover, ^1^H NMR spectra of pyrrolo[2,3-*b*]quinoxalines 3a–e all showed two doublets of doublets (dd) at chemical shifts around 8.41 ppm and 8.05 ppm, representing two protons at the 5,8-positions of the quinoxaline moieties. In addition, resonance signals of two methylene protons and three methyl protons of the ethoxycarbonyl group (–C(O)OCH_2_CH_3_) in compounds 3a–e were characterized by a quartet (q) and a triplet (t) at chemical shifts around 4.36 ppm and 1.26 ppm, respectively.

### 3.2. X-ray study of ethyl 1,2-diphenyl-1*H*-pyrrolo[2,3-*b*]quinoxaline-3-carboxylate (3a)

**Fig. 2:**
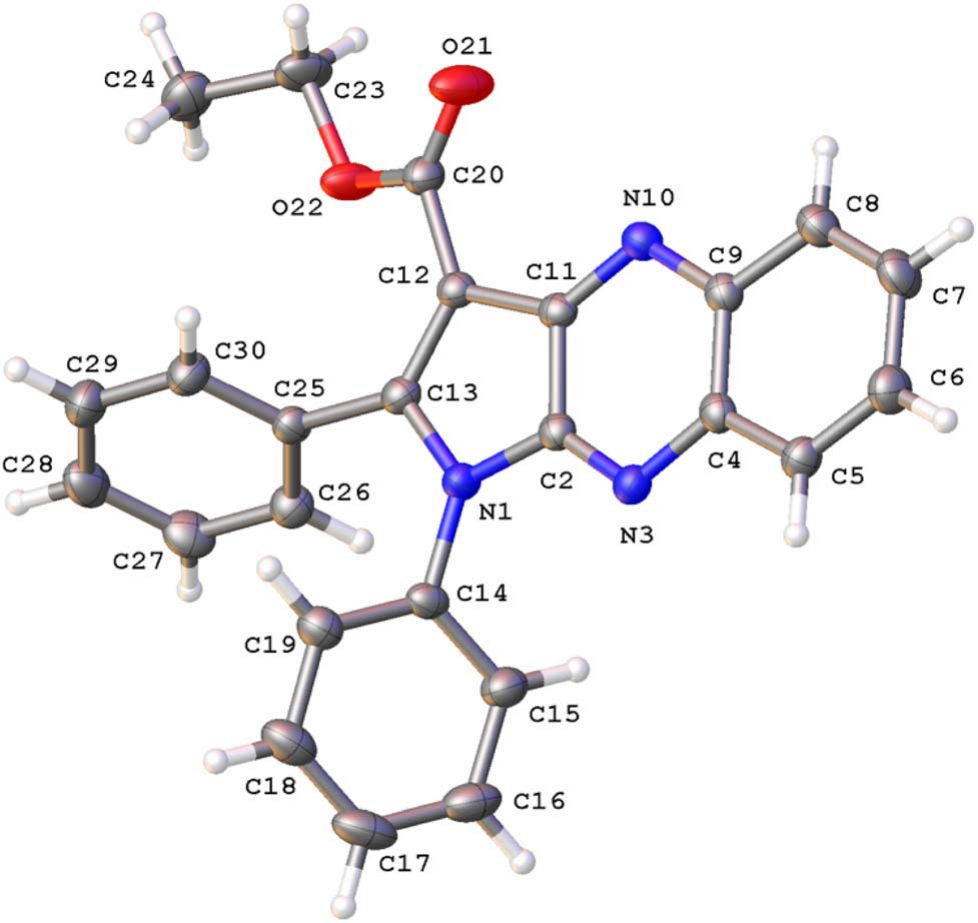
A view of the molecular structure of 3a, with atom labels and displacement ellipsoids drawn at the 30% probability level. H atoms are shown as small circles of arbitrary radii.

The structure of 3a was verified through single-crystal X-ray diffraction (Fig. 2). The central pyrrolo[2,3-*b*]quinoxaline ring is planar (r.m.s. deviation 0.039 Å) and makes an angle of 57.83(13)° with phenyl ring C14–C19 and of 63.63(13)° with phenyl ring C25–C30.

Both phenyl rings make a mutual angle of 57.21(17)°. The best plane through ester group C12, C20, O21, O22 makes an angle of 18.13(14)° with the central ring system. The crystal packing of 3a (Fig. S1†) is characterized by C17–H17⋯O21^i^ hydrogen bonds resulting in chain formation along the *c* direction [C17–H17: 0.93 Å, H17⋯O21: 2.35 Å, C17⋯O21: 3.383(5) Å, C17–H17⋯O21: 150°; symmetry code: (i) 3/2 − *x*, 1 − *y*, 1/2 + *z*]. Neighboring chains connect by C–H⋯π interactions between C24–H24A and the central pyrazine ring [H24A⋯Cg^ii^: 2.81 Å; Cg is the centroid of ring N3, C4, C9, N10, C11, C2; symmetry code: (ii) 1 − *x*, −1/2 + *y*, 1/2 − *z*].

### 3.3. The radical scavenging activity of pyrrolo[2,3-*b*]quinoxalines 3a–e

#### 3.3.1. DPPH antioxidant assay

To assess the antioxidant activity of the synthetic pyrrolo[2,3-*b*]quinoxalines, an initial evaluation was conducted using the DPPH assay, which was based on the literature,^26^ using quercetin as the standard antioxidant (Table 3). The compound under investigation demonstrated a reduced DPPH scavenging activity (0.283–0.325 mM) compared to quercetin (9.97 ± 0.25 mg mL^−1^, 0.033 mM) under the evaluated conditions. The synthetic compound 3a (24% DPPH at 128 mg mL^−1^) exhibited the most effective DPPH antiradical activity. This performed over twofold better than 3b (DPPH: 7%) or 3d (DPPH: 9%). 3c and 3e exhibited intermediate activities at 20% and 18% DPPH, respectively. Nevertheless, prior research has demonstrated that pyrrolo[2,3-*b*]quinoxaline derivatives possess significant radical scavenging capabilities in contexts that are more biologically significant, such as lipid peroxidation or against HO˙ radicals.^47–49^ Consequently, computational chemistry has been employed to further examine the radical (*i.e.*, HO˙ and HOO˙) scavenging activity of the most active compound (3a) in physiological environments.

**Table 3:** DPPH radical scavenging activity

Compound	DPPH (%)				EC_50_ (μg mL^−1^)
	265 (μg mL^−1^)	128 (μg mL^−1^)	32 (μg mL^−1^)	8 (μg mL^−1^)	
3a	30	24	0	0	>128
3b	9	7	0	0	>128
3c	23	20	0	0	>128
3d	10	9	0	0	>128
3e	21	18	0	0	>128
Quercetin	100	100	45.5	0	9.97 ± 0.25

#### 3.3.2. Calculations of the HO˙/HOO˙ radical scavenging activity of the most active compound (3a)

##### (a) Structure evaluation

**Fig. 3:**
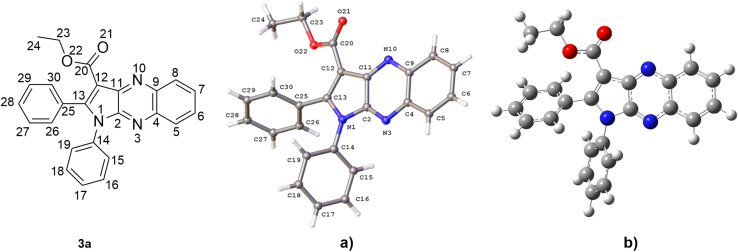
Crystal (a) and optimized (b) structures of 3a.

The hexagon rings of 3a are capable of undergoing rotation in order to generate an extensive range of conformers. Since the conformer with the highest probability of engaging in a radical scavenging reaction is also the most stable, the initial step involved assessing the electron energy levels of every conceivable conformers of 3a.^50^ Following this, the conformer exhibiting the least amount of electronic energy was optimized utilizing the M06-2X/6-311++G (d,p) level of theory. The resulting data, including the angles (°) and bond length (Å), was subsequently compared to the crystal structure and is displayed in Fig. 3, S19†, Table S2 and ESI.† It was found that the deviations in bond lengths and angles between the optimized and crystal structures were less than 0.025 Å and 2°, *i.e.* less than 1.7% and 1.6%, respectively. This internally confirms the high accuracy of the model chemistry used in this work.

##### (b) The antiradical activity in the gas phase

To identify the primary mechanisms by which 3a would function under the more complex physiological conditions, its antioxidant properties were initially evaluated in the gas phase. Depending on the molecular structures involved, various pathways may be followed by the 3a − HO˙/HOO˙ reactions. These are formal hydrogen transfer (FHT), radical adduct formation (RAF), and single electron transfer (SET) pathways.^33,36,38,51–54^ To identify the preferred radical quenching mechanism (FHT, SET, and RAF) followed by 3a to eliminate the HO˙ and HOO˙ radicals, first the gas phase Gibbs free energy changes (Δ*G*°) were computed. Table 4 displays the results.

**Table 4:** Calculated Δ*G*° values (in kcal mol^−1^) of the reactions of 3a with HO˙ following the FHT and RAF mechanisms

Mechanisms	Positions	HO˙	HOO˙
FHT	C23–H	−21.7	9.6
	C24–H	−16.4	14.8
RAF	C2	−5.0	
	C4	4.2	
	C5	−19	
	C6	−9.3	
	C7	−10.9	
	C8	−15.5	
	C9	1.5	
	C11	−6.9	
	C12	−20.7	
	C13	−24.5	
	C14	−9.5	
	C15	−10.2	
	C16	−6.1	
	C17	−7.2	
	C18	−5.2	
	C19	−9.8	
	C25	−2.7	
	C26	−8.3	
	C27	−5.8	
	C28	−8.2	
	C29	−5.5	
	C30	−9.4	
	N3	13.8	
	N10	−2.5	
SET		155.6	165.5

**Fig. 4:**
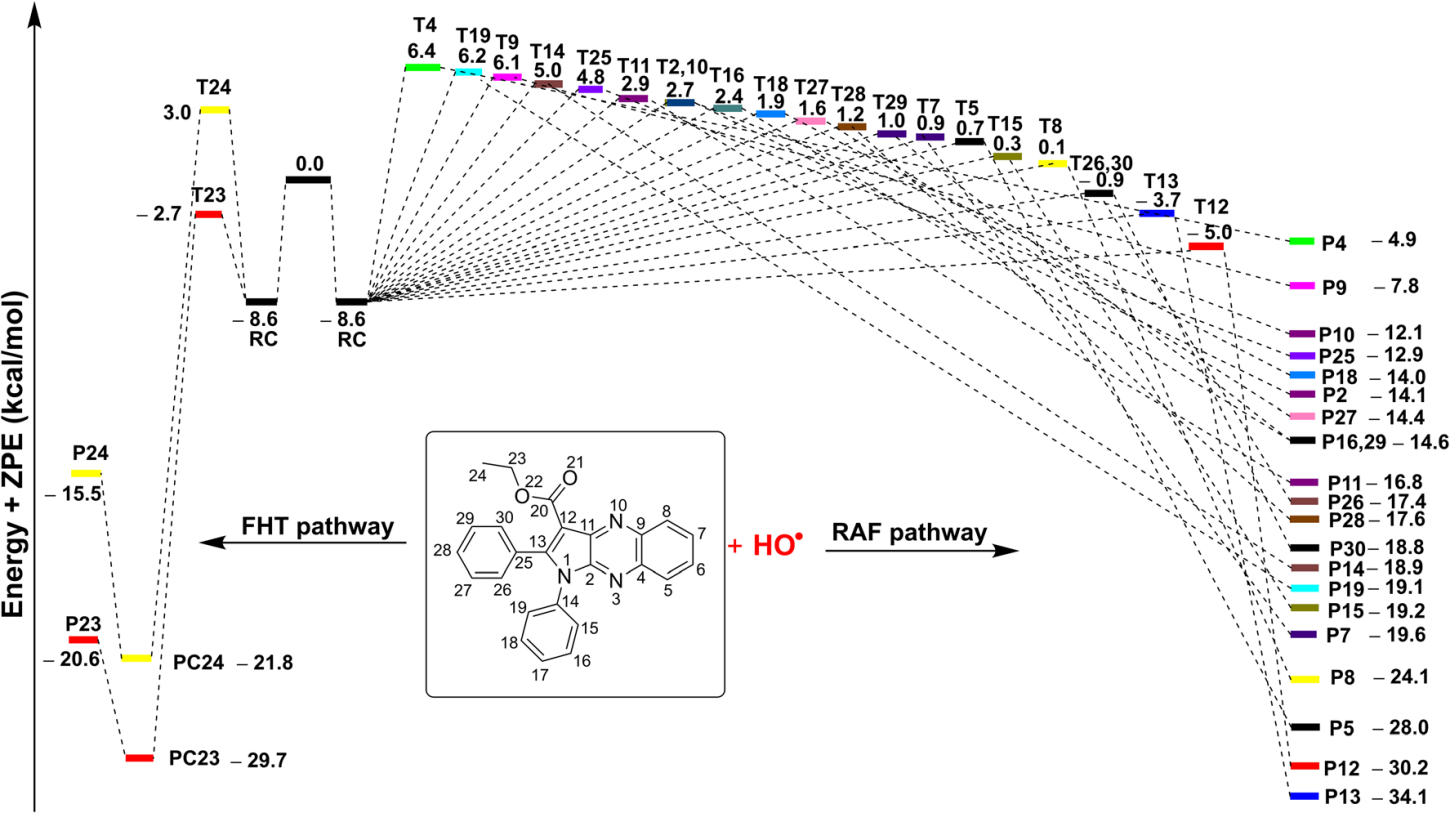
The potential energy surfaces (PES) of the 3a + HO˙ according to FHT and RAF reactions (RC: pre-complex; TS: transition state; PC: post-complex; P: product).

**Fig. 5:**
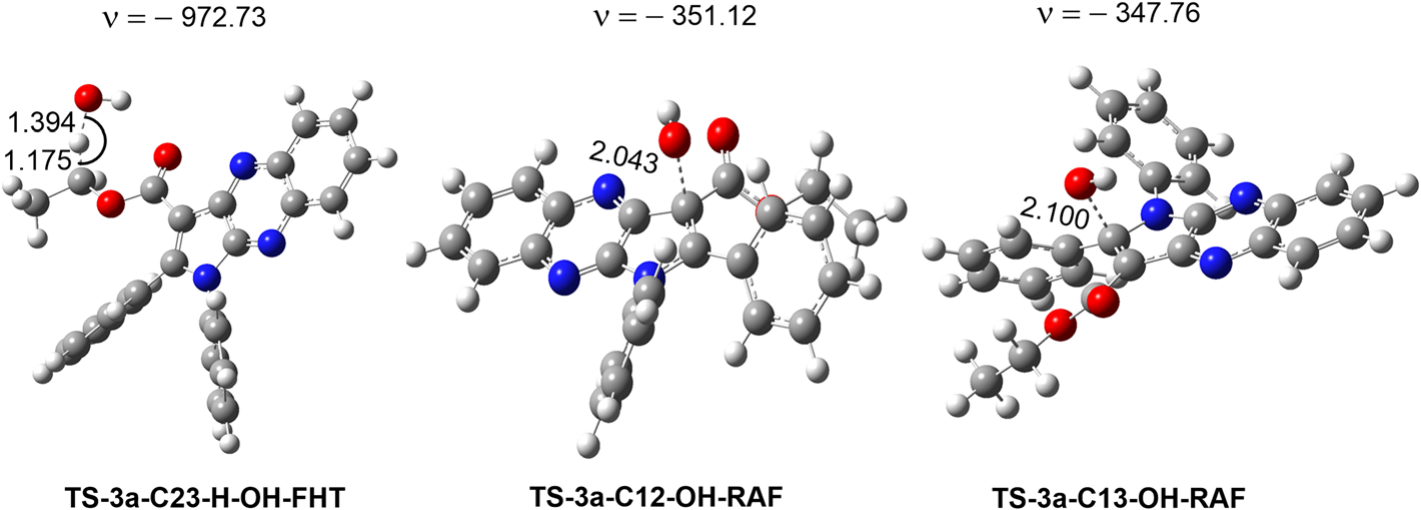
Optimized geometries of the main transition states between 3a and HO˙ radicals according to FHT and RAF processes.

The radical scavenging activity for the HO˙ radicals are thermodynamically spontaneous (Δ*G*° < 0) at most positions in 3a, with the exception of the SET reaction (Δ*G*° = 155.6 kcal mol^−1^) and the RAF reactions at C4, C9, and N13 (Δ*G*° > 0). On the contrary, the radical scavenging of the HOO˙ radical is not spontaneous in any of the mechanisms that have been investigated. Thus, the radical scavenging of HOO˙ was excluded from the investigation. The kinetic evaluation of the possible positions (Δ*G*° < 0, Table 4) was conducted in a consistent manner to assess the scavenging of HO˙ radical by 3a in the gas phase. The outcomes of this analysis are displayed in Table 5, Fig. 4 and 5.

**Table 5:** Calculated activation energies Δ*G*^‡^ (kcal mol^−1^), tunneling corrections (*κ*), *k*_Eck_, *k*_overall_ (M^−1^ s^−1^) and branching ratios *Γ* (%) at 298.15 K for the HO˙ scavenging of 3a

Mechanisms	Positions	Δ*G*^‡^	*κ*	*k*_Eck_	*Γ*
FHT	C23	4.1	2.5	4.94 × 10^10^	18.3
	C24	11.0	5.2	7.83 × 10^5^	0.0
RAF	C2	10.1	1.5	3.67 × 10^5^	0.0
	C4	13.4	1.4	1.26 × 10^3^	0.0
	C5	7.2	1.2	3.98 × 10^7^	0.0
	C6	7.6	1.2	1.87 × 10^7^	0.0
	C7	7.3	1.2	3.31 × 10^7^	0.0
	C8	6.5	1.2	1.23 × 10^8^	0.0
	C9	13.1	1.4	2.35 × 10^3^	0.0
	C11	10.0	1.4	4.04 × 10^5^	0.0
	C12	2.3	1.2	1.45 × 10^11^	53.5
	C13	2.8	1.3	7.60 × 10^10^	28.1
	C14	12.7	1.5	4.52 × 10^3^	0.0
	C15	6.8	1.0	6.63 × 10^7^	0.0
	C16	8.8	1.3	3.13 × 10^6^	0.0
	C18	8.4	1.4	6.63 × 10^6^	0.0
	C19	13.4	1.4	1.14 × 10^3^	0.0
	C25	12.7	1.3	3.85 × 10^3^	0.0
	C26	6.8	1.2	7.95 × 10^7^	0.0
	C27	9.0	1.3	2.05 × 10^6^	0.0
	C28	7.4	1.3	3.01 × 10^7^	0.0
	C29	8.0	1.3	1.02 × 10^7^	0.0
	C30	6.8	1.2	7.95 × 10^7^	0.0
	N10	10.3	1.7	2.95 × 10^5^	0
***k***_**overall**_				**2.70 × 10** ^**11**^	

The results presented in the PES (Fig. 4) indicate that the C12 position has the lowest reaction barrier value of 3.6 kcal mol^−1^. In contrast, the C4 position of 3a displayed the highest reaction barrier value of 15.0 kcal mol^−1^. The FHT reaction of C23–H and the RAF at position C13 exhibited the second and third lowest reaction barriers, measuring 5.9 and 4.9 kcal mol^−1^, respectively. In contrast, the 3a + HO˙ reaction has comparatively lower reaction barriers, ranging from 7.7 to 15.0 kcal mol^−1^ in the rest of the reactions. According to the PES analysis, the HO˙ radical scavenging activity of 3a is dominated by the FHT reaction at the C23–H bond and the RAF reaction at the C12/13.

Table 5 indicates that the overall rate constant (*k*_overall_) for the gas phase scavenging of the HO˙ radical was 2.70 × 10^11^ M^−1^ s^−1^. The rate constant for the 3a + HO˙ reaction was determined by the RAF reaction at positions C12 and C13, with rate constants of 1.45 × 10^11^ (*Γ* = 53.5%) and 7.60 × 10^10^ M^−1^ s^−1^ (*Γ* = 28.1%), respectively, while the H-abstraction contributed approximately 18.2% (*k*_Eck_ = 4.94 × 10^10^ M^−1^ s^−1^) to the overall rate constant. This result is consistent with the thermodynamic and the PES analysis (Table 4 and Fig. 4), which determined that the RAF-C12/13 and FHT-C23 reactions had the lowest Δ*G*° values (Δ*G*° = −20.7, −24.5 and −21.7 kcal mol^−1^ for C12, C13 and C23, respectively). No other reaction pathways make any notable contributions.

##### (c) The antiradical activity in the lipid medium

Previous studies established that pyrrolo[2,3-*b*]quinoxaline derivatives have good radical scavenging activity in biologically consequential contexts, including lipid peroxidation and resistance to HO˙ radicals.^47–49^ Therefore, the QM-ORSA protocol was employed here to assess the kinetics of the HO˙-scavenging reactions taking place in the lipid medium following the main reactions in nonpolar media *i.e.*, the RAF reaction at C12/13 and FHT reaction in the C23–H bond. The findings are presented in Table 6.

**Table 6:** The calculated Δ*G*^‡^ (kcal mol^−1^), *k*_app_, *k*_overall_ (M^−1^ s^−1^) and *Γ* (%) at 298.15 K, in the 3a oxidation by HO˙ radicals in the lipid medium

Mechanisms		Pentyl ethanoate			
		Δ*G*^‡^	*κ*	*k*_app_	*Γ*
FHT	C23–H	6.0	2.1	4.10 × 10^8^	47.9
RAF	C12	5.6	1.1	4.40 × 10^8^	51.4
	C13	8.3	1.1	5.60 × 10^6^	0.7
***k***_**overall**_				**8.56 × 10** ^**8**^	

The *k*_overall_ for the 3a + HO˙ reaction in pentyl ethanoate was 8.56 × 10^8^ M^−1^ s^−1^. The C12 position RAF mechanism (*Γ* = 51.4%) and the FHT pathway at the C23–H bond (*Γ* = 47.9%) were found to define the HO˙ antiradical activity. In contrast, the C13 position RAF reaction accounted for a mere 0.7% of the overall reaction. The hydroxyl radical scavenging capability of 3a in a non-polar environment is analogous to that of widely used antioxidants including Trolox, melatonin,^55^ indole-3-carbinol,^56^ and gallic acid.^57^3a is therefore an effective hydroxyl radical scavenger in lipid environments.

## 4. Conclusion

Successful synthesis of five pyrrolo[2,3-*b*]quinoxaline derivatives was achieved. 3a demonstrates the greatest DPPH radical scavenging activity, as determined by the DPPH assay. The structure of the synthesized lead compound 3a was verified by X-ray analysis. In pentyl ethanoate, the thermodynamic and kinetic calculations also revealed that 3a exhibited HO radical scavenging activity with *k*_overall_ = 8.56 × 10^8^ M^−1^ s^−1^. In contrast, in nonpolar media, 3a demonstrated only a negligible capacity to scavenge hydroperoxyl radicals. The capacity of 3a to scavenge hydroxyl radicals in non-polar environments is comparable to that of conventional antioxidants including Trolox, melatonin, indole-3-carbinol, and gallic acid. Therefore in physiological lipid environments 3a holds potential as a HO˙ radical scavenger.

## Data availability

The data supporting this article have been included as part of the ESI.† Crystallographic data for [3a] has been deposited at the [CCDC] under [2310877].

## Conflicts of interest

There are no conflicts to declare.

## Supplementary Material

RA-014-D4RA03108C-s001

RA-014-D4RA03108C-s002
