# Supplementary material for: Synthesis and computational evaluation of the antioxidant activity of pyrrolo[2,3-b]quinoxaline derivatives
Source: RSC Adv. 2024 Aug 5;14(34):24438–46. doi: 10.1039/d4ra03108c (PMC11299642; doi:10.1039/d4ra03108c)
Supplement: RA-014-D4RA03108C-s001 [file RA-014-D4RA03108C-s001.pdf]

Supplementary Information (SI) for RSC Advances.
This journal is © The Royal Society of Chemistry 2024

Supporting Information (SI)

Synthesis and computational evaluation of the antioxidant activity of pyrrolo[2,3-*b*]quinoxaline derivatives

Nguyen Tran Nguyen[1*], Vo Viet Dai[1], Adam Mechler[2], Luc Van Meervelt[3], Nguyen Thi Hoa[4] and Quan V. Vo[4*]

[1]*The University of Danang-University of Science and Education, Danang 550000, Vietnam.*
[2]*Department of Biochemistry and Chemistry, La Trobe University, Victoria 3086, Australia.*
[3]*Biomolecular Architecture, Department of Chemistry, KU Leuven, Celestijnenlaan 200F, B-3001 Leuven, Belgium*
[4]*The University of Danang - University of Technology and Education, Danang 550000, Vietnam.*

**Corresponding authors: ntnguyen@ued.udn.vn; vvquan@ute.udn.vn*

Table of Contents

Table S1. The method to calculate rate constant following the conventional transition state theory S3

Figure S1. ^{1}H NMR spectrum of ethyl 1,2-diphenyl-1H-pyrrolo[2,3-*b*]quinoxaline-3-carboxylate (3a) S5

Figure S2. ^{13}C NMR spectrum of ethyl 1,2-diphenyl-1H-pyrrolo[2,3-*b*]quinoxaline-3-carboxylate (3a) S5

Figure S3. ESI-HRMS spectrum of ethyl 1,2-diphenyl-1H-pyrrolo[2,3-*b*]quinoxaline-3-carboxylate (3a) S6

Figure S4. ^{1}H NMR spectrum of ethyl 1,2-diphenyl-2,4-dihydro-1H-pyrrolo[2,3-*b*]quinoxaline-3-carboxylate (3a') S6

Figure S5. ^{13}C NMR spectrum of ethyl 1,2-diphenyl-2,4-dihydro-1H-pyrrolo[2,3-*b*]quinoxaline-3-carboxylate (3a') S7

Figure S6. ESI-HRMS spectrum of ethyl 1,2-diphenyl-2,4-dihydro-1H-pyrrolo[2,3-*b*]quinoxaline-3-carboxylate (3a') S7

Figure S7. ^{1}H NMR spectrum of ethyl 1-(3-nitrophenyl)-2-(p-tolyl)-1H-pyrrolo[2,3-*b*]quinoxaline-3-carboxylate (3b) S8

Figure S8. ^{13}C NMR spectrum of ethyl 1-(3-nitrophenyl)-2-(p-tolyl)-1H-pyrrolo[2,3-*b*]quinoxaline-3-carboxylate (3b) S8

Figure S9. ESI-HRMS spectrum of ethyl 1-(3-nitrophenyl)-2-(p-tolyl)-1H-pyrrolo[2,3-*b*]quinoxaline-3-carboxylate (3b) S9

Figure S10. ^{1}H NMR spectrum of ethyl 2-(4-nitrophenyl)-1-phenyl-1H-pyrrolo[2,3-*b*]quinoxaline-3-carboxylate (3c) S10

Figure S11. ^{13}C NMR spectrum of ethyl 2-(4-nitrophenyl)-1-phenyl-1H-pyrrolo[2,3-*b*]quinoxaline-3-carboxylate (3c) S10

Figure S12. ESI-HRMS spectrum of ethyl 2-(4-nitrophenyl)-1-phenyl-1H-pyrrolo[2,3-*b*]quinoxaline-3-carboxylate (3c) S10

Figure S13. ^{1}H NMR spectrum of ethyl 1-phenyl-2-(p-tolyl)-1H-pyrrolo[2,3-*b*]quinoxaline-3-carboxylate (3d) S11

Figure S14. ^{13}C NMR spectrum of ethyl 1-phenyl-2-(p-tolyl)-1H-pyrrolo[2,3-*b*]quinoxaline-3-carboxylate (3d) S11

Figure S15. ESI-HRMS spectrum of ethyl 1-phenyl-2-(p-tolyl)-1H-pyrrolo[2,3-*b*]quinoxaline-3-carboxylate (3d) S12

Figure S16. ^{1}H NMR spectrum of ethyl 1-benzyl-2-(4-nitrophenyl)-1H-pyrrolo[2,3-*b*]quinoxaline-3-carboxylate (3e) S12

Figure S17. ^{13}C NMR spectrum of ethyl 1-benzyl-2-(4-nitrophenyl)-1H-pyrrolo[2,3-*b*]quinoxaline-3-carboxylate (3e) S13

Figure S18. ESI-HRMS spectrum of ethyl 1-benzyl-2-(4-nitrophenyl)-1H-pyrrolo[2,3-*b*]quinoxaline-3-carboxylate (3e) S13

Figure S19. Partial crystal packing of X showing the chain formation along the *c* direction by C-H...O hydrogen bonding and C-H...π interactions. S14

Table S2. Crystal data and structure refinement details for 3a S15

Table S3: The Cartesian coordinates and energies of TS of the reaction between 4b with HO$^{\bullet}$ and HOO$^{\bullet}$ in the studied environments. S17

References 44

Table S1. The method to calculate rate constant following the conventional transition state theory

The rate constant (k) was calculated by using the conventional transition state theory (TST) (at 298.15 K, 1M standard state) according to the equation (1):

$$k = \sigma\kappa\frac{k_B T}{h}e^{-(\Delta G^{\neq})/RT} \qquad (1)$$

Where: σ is the reaction symmetry number ,[1,2]

κ contains the tunneling corrections calculated using the Eckart barrier,[3]

k_B is the Boltzmann constant,

h is the Planck constant,

$\Delta G^{\neq}$ is the Gibbs free energy of activation.

The Marcus Theory was used to estimate the reaction barriers of SET reactions.[4-7] The free energy of reaction $\Delta G^{\neq}$ for the SET pathway was computed following the equations (2,3).

$$\Delta G^{\neq}_{SET} = \frac{\lambda}{4}\left(1 + \frac{\Delta G^0_{SET}}{\lambda}\right)^2 \qquad (2)$$

$$\lambda \approx \Delta E_{SET} - \Delta G^0_{SET} \qquad (3)$$

where ΔG_{SET} is the Gibbs energy of reaction, ΔE_{SET} is the non-adiabatic energy difference between reactants and vertical products for SET.[8,9]

For rate constants that were close to the diffusion limit a correction was applied to yield realistic results[10]. The apparent rate constants (k_{app}) were calculated following the Collins–Kimball theory in the solvents at 298.15K;[11] the steady-state Smoluchowski rate constant (k_D) for an irreversible bimolecular diffusion–controlled reaction was calculated following the literature as corroding to equations (4,5).[10,12]

$$k_{app} = \frac{k_{TST}k_D}{k_{TST} + k_D} \qquad (4)$$

$$k_D = 4\pi R_{AB}D_{AB}N_A \qquad (5)$$

where R_{AB} is the reaction distance, N_A is the Avogadro constant, and $D_{AB} = D_A + D_B$ (D_{AB} is the mutual diffusion coefficient of the reactants A and B),[11,13] where D_A or D_B is estimated using the Stokes–Einstein formulation (6).[14,15]

$$D_{A\ or\ B} = \frac{k_B T}{6\pi\eta a_{A\ or\ B}} \qquad (6)$$

η is the viscosity of the solvents (η(pentyl ethanoate) = 8.62×10^{-4} Pa s) and a is the radius of the solute.

The kinetic study requires different considerations. Pentyl ethanoate (ε = 4.73) are the *de facto* standard solvents in the literature to mimic the nonpolar environments in the human body.[10,16-18] Thus, this solvent was used to model the physiological environments. The solvent cage effects were included following the corrections proposed by Okuno,[19] adjusted with the free volume theory according to the Benson correction[10,20-22] to reduce over-penalizing entropy losses in solution. For the species that have multiple conformers, all of these were investigated and the conformer with the lowest electronic energy was included in the analysis.[17,18] The hindered internal rotation treatment was also applied to the single bonds to ensure that the obtained conformer has the lowest electronic energy.[18,23] All transition states were characterized by the existence of only one single imaginary frequency. Intrinsic coordinate calculations (IRCs) were performed to ensure that each transition state is connected correctly with the pre-complex and post-complex.

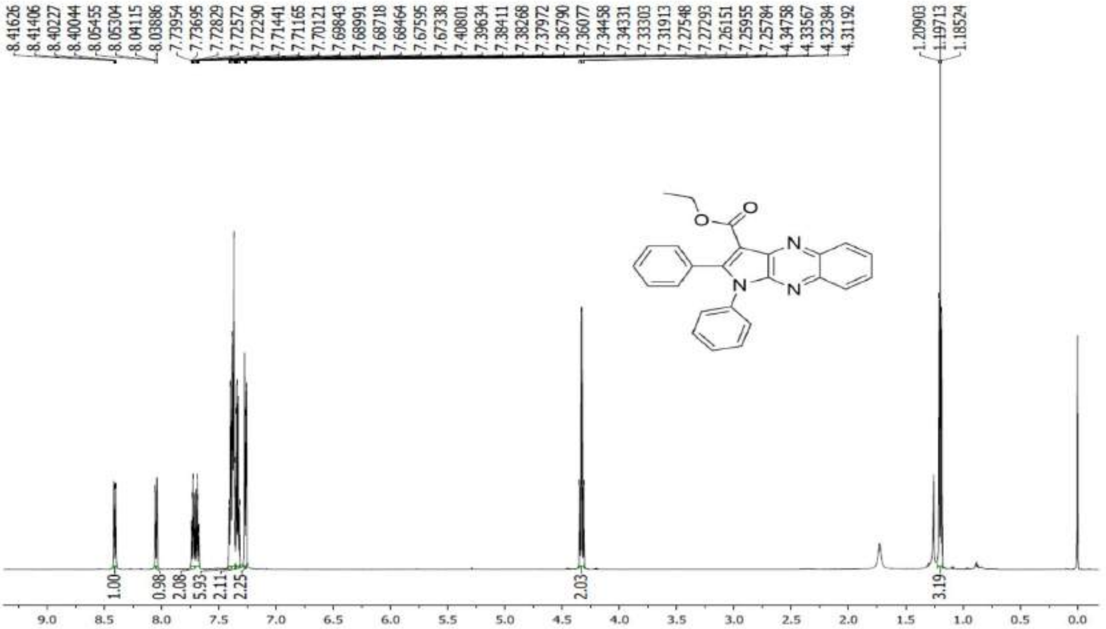

Figure S1. ^{1}H NMR spectrum of ethyl 1,2-diphenyl-1H-pyrrolo[2,3-*b*]quinoxaline-3-carboxylate (3a)

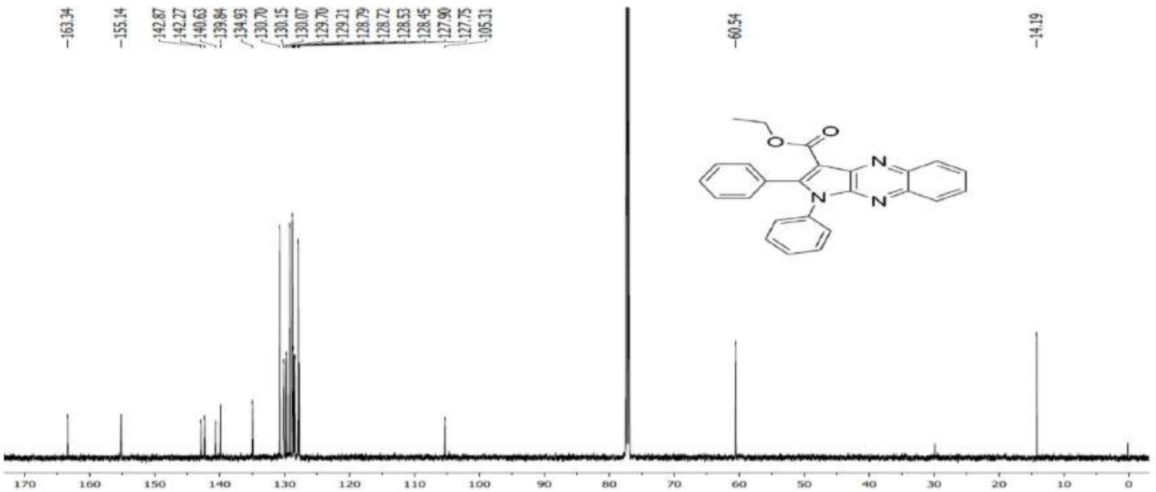

Figure S2. ^{13}C NMR spectrum of ethyl 1,2-diphenyl-1H-pyrrolo[2,3-*b*]quinoxaline-3-carboxylate (3a)

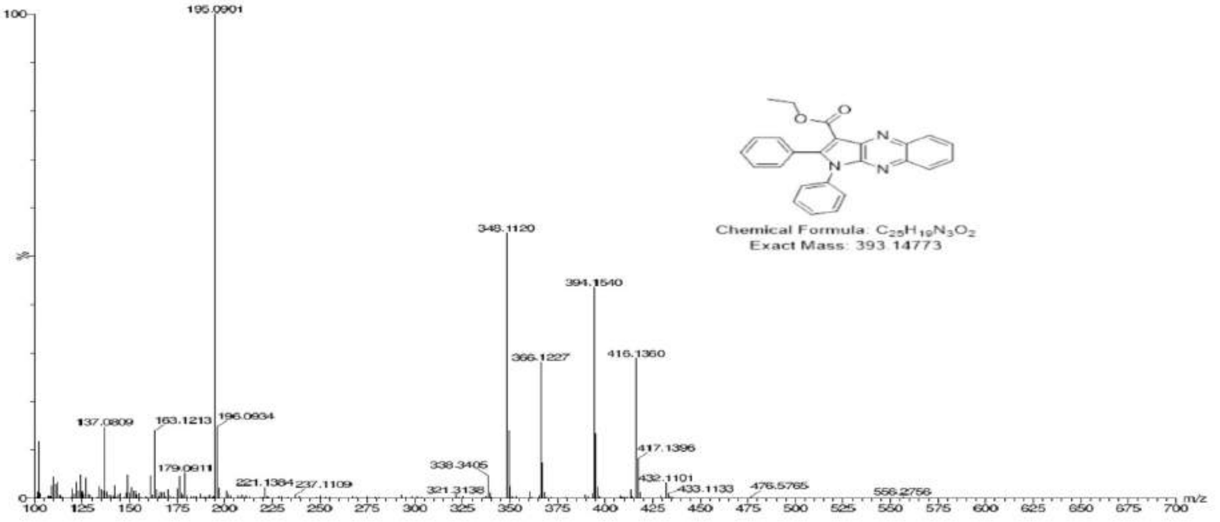

Figure S3. ESI-HRMS spectrum of ethyl 1,2-diphenyl-1H-pyrrolo[2,3-*b*]quinoxaline-3-carboxylate (3a)

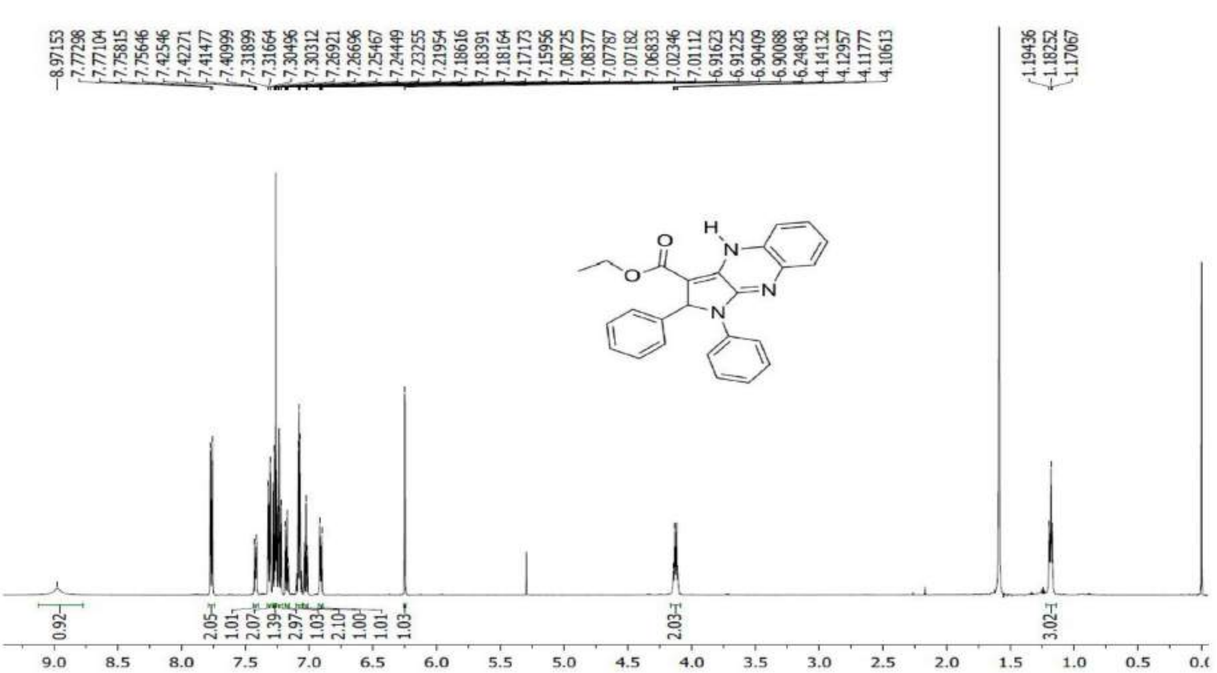

Figure S4. ^{1}H NMR spectrum of ethyl 1,2-diphenyl-2,4-dihydro-1H-pyrrolo[2,3-*b*]quinoxaline-3-carboxylate (3a')

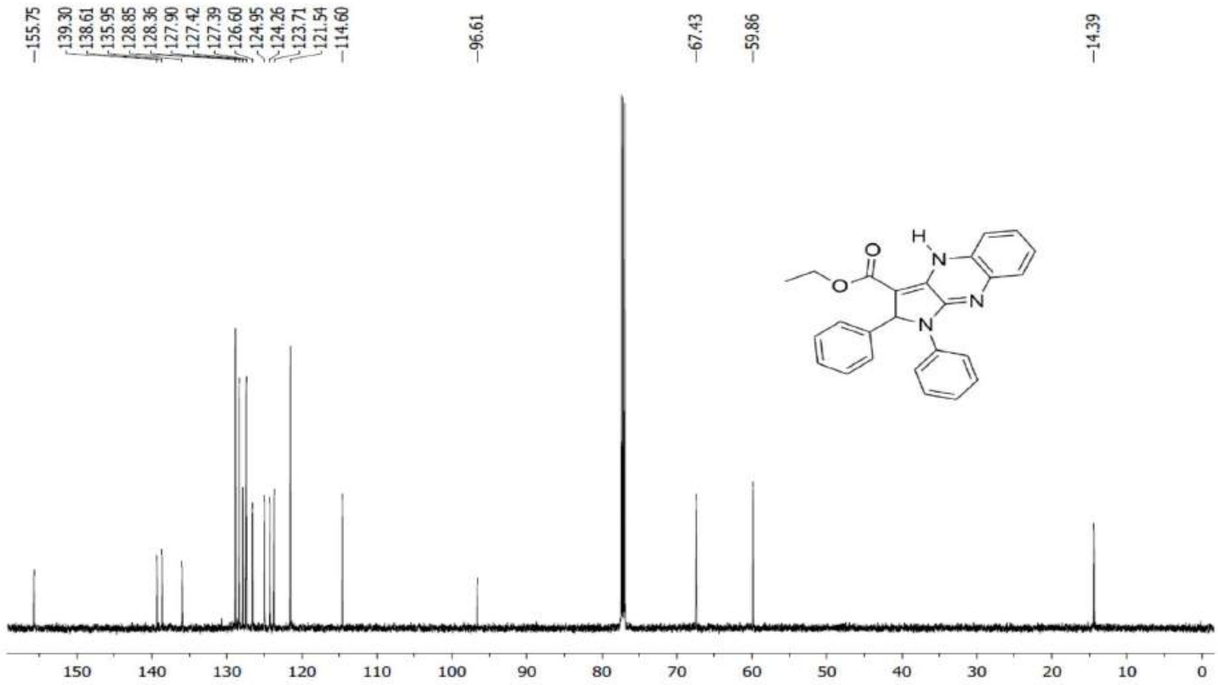

Figure S5. ^{13}C **NMR spectrum of ethyl 1,2-diphenyl-2,4-dihydro-1H-pyrrolo[2,3-*b*]quinoxaline-3-carboxylate (3a')**

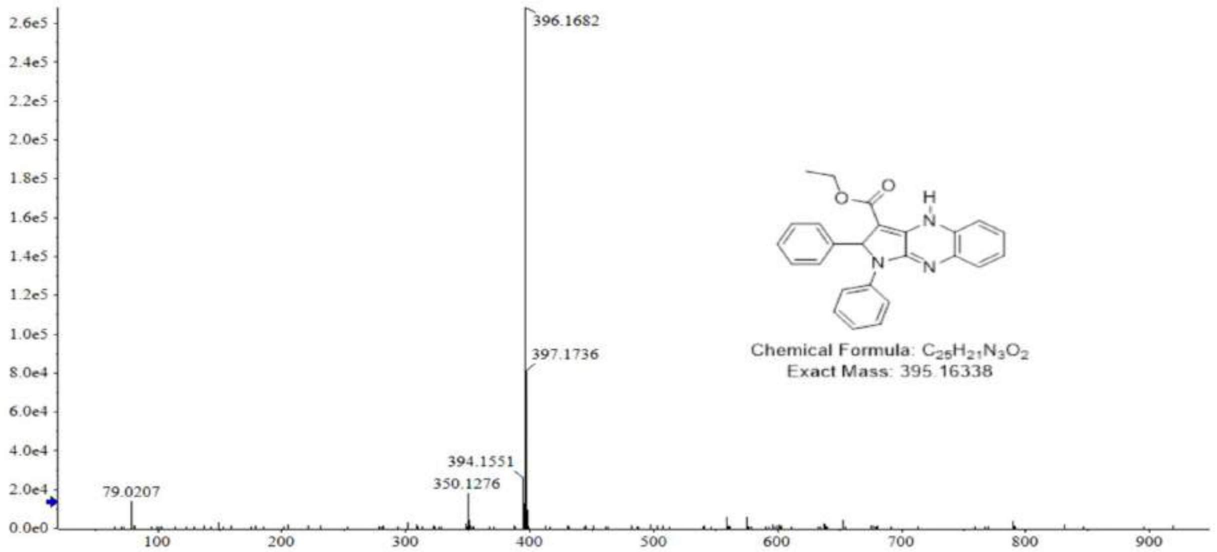

Figure S6. ESI-HRMS spectrum of ethyl 1,2-diphenyl-2,4-dihydro-1H-pyrrolo[2,3-*b*]quinoxaline-3-carboxylate (3a')

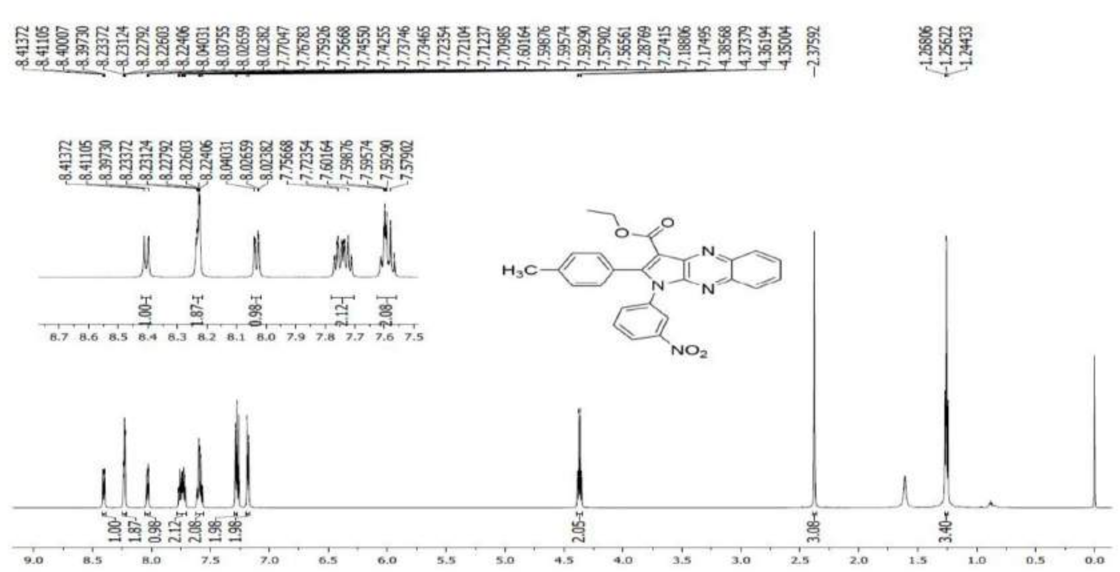

Figure S7. **^{1}H NMR spectrum of ethyl 1-(3-nitrophenyl)-2-(p-tolyl)-1H-pyrrolo[2,3-*b*]quinoxaline-3-carboxylate (3b)**

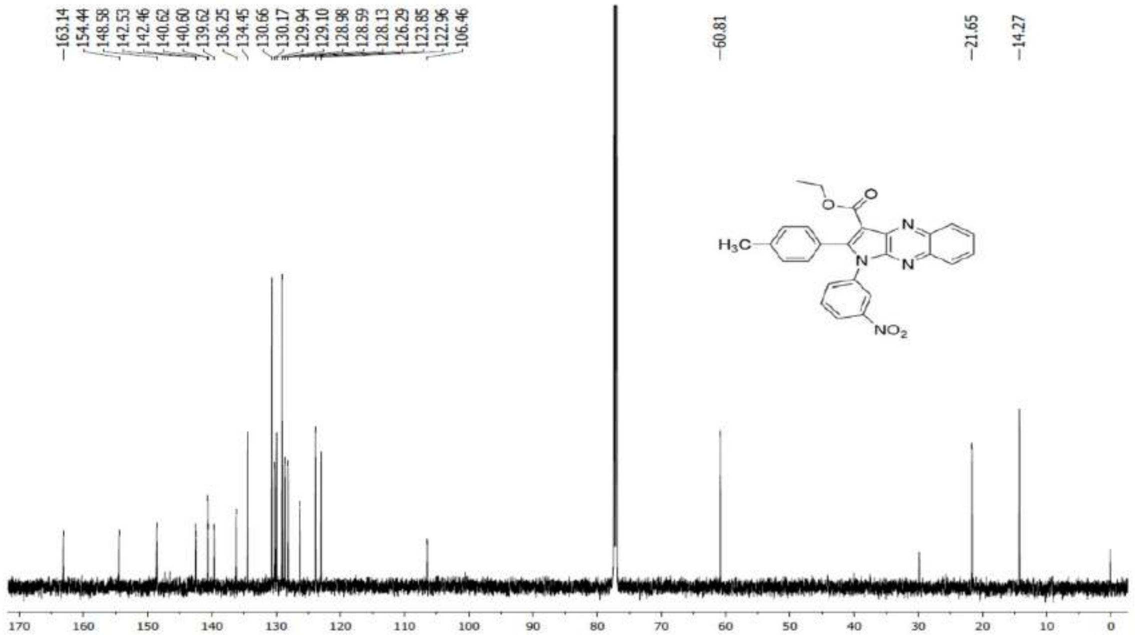

Figure S8. **^{13}C NMR spectrum of ethyl 1-(3-nitrophenyl)-2-(p-tolyl)-1H-pyrrolo[2,3-*b*]quinoxaline-3-carboxylate (3b)**

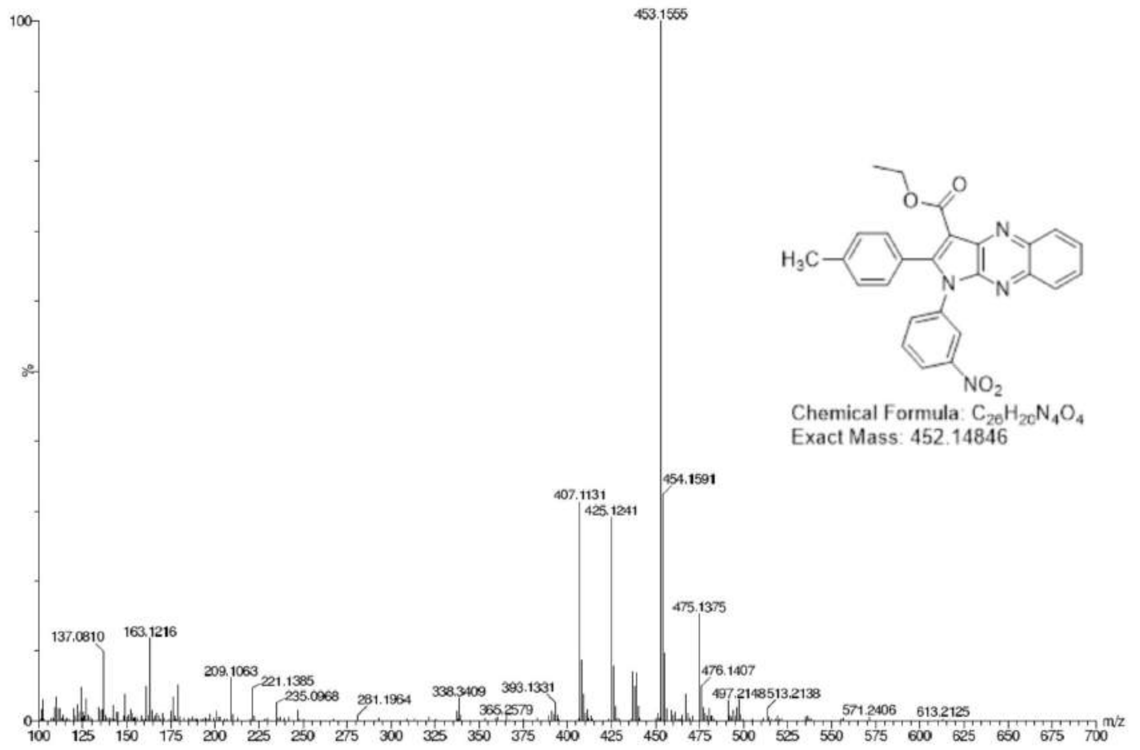

Figure S9. ESI-HRMS spectrum of ethyl 1-(3-nitrophenyl)-2-(p-tolyl)-1H-pyrrolo[2,3-*b*]quinoxaline-3-carboxylate (3b)

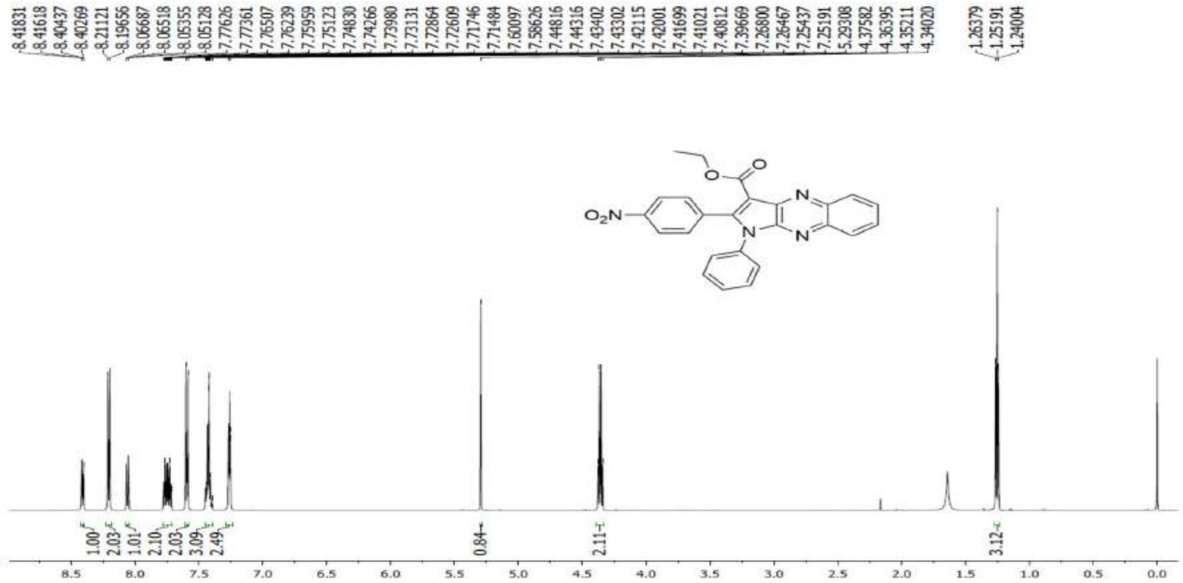

Figure S10. ^{1}H **NMR spectrum of ethyl 2-(4-nitrophenyl)-1-phenyl-1H-pyrrolo[2,3-*b*]quinoxaline-3-carboxylate (3c)**

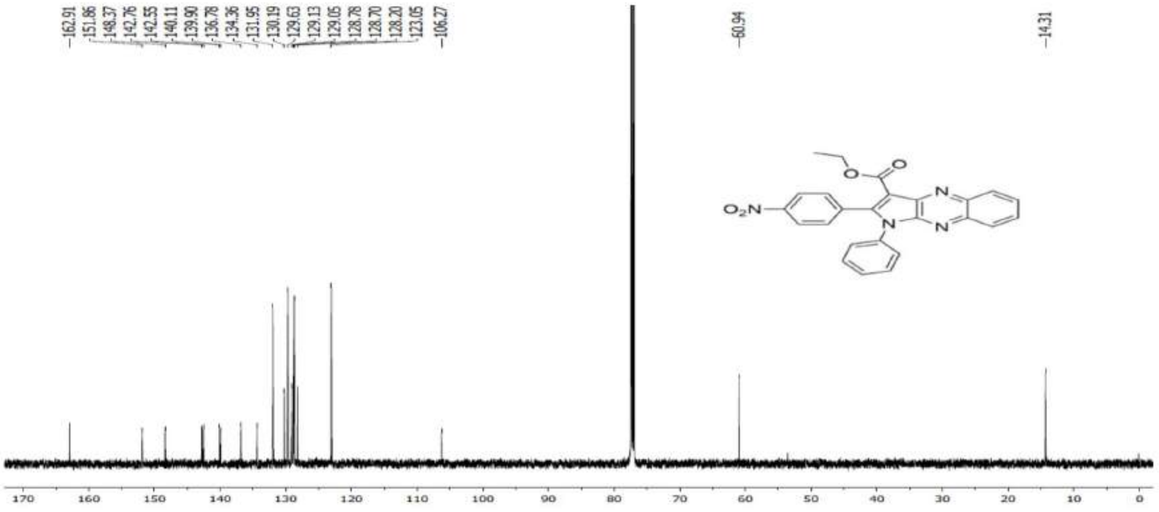

Figure S11. ^{13}C **NMR spectrum of ethyl 2-(4-nitrophenyl)-1-phenyl-1H-pyrrolo[2,3-*b*]quinoxaline-3-carboxylate (3c)**

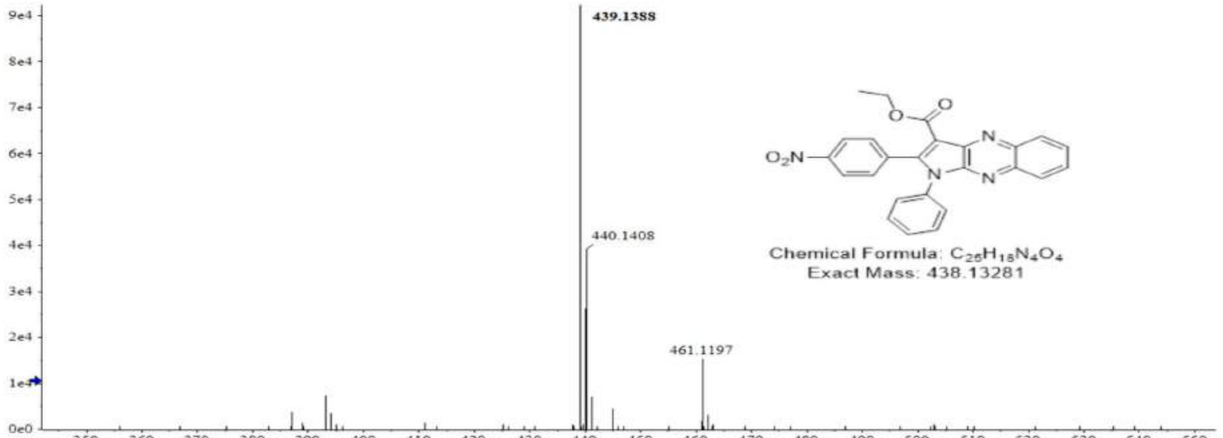

Figure S12. ESI-HRMS spectrum of ethyl 2-(4-nitrophenyl)-1-phenyl-1H-pyrrolo[2,3-*b*]quinoxaline-3-carboxylate (3c)

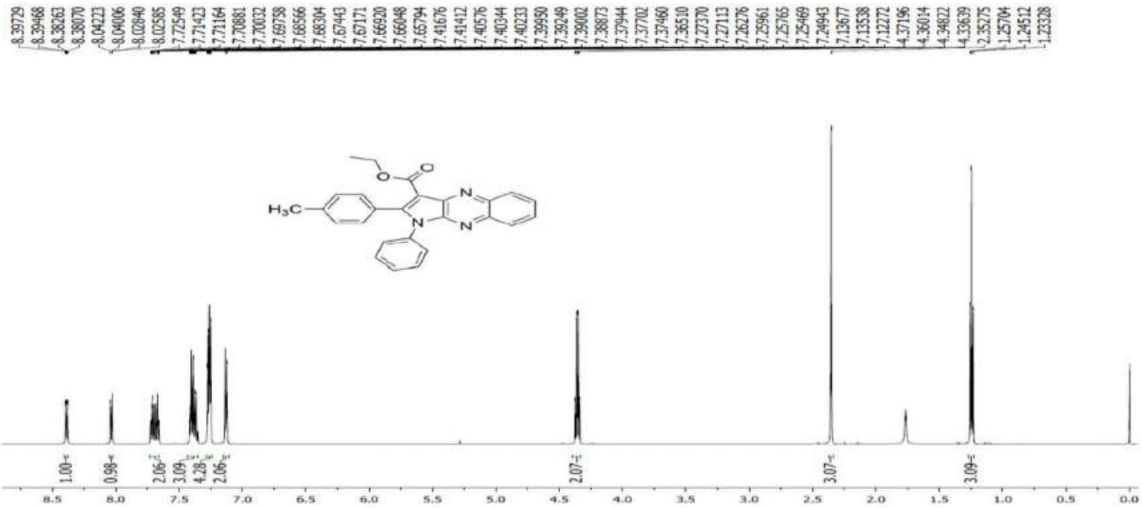

Figure S13. ^{1}H **NMR spectrum of ethyl 1-phenyl-2-(p-tolyl)-1H-pyrrolo[2,3-*b*]quinoxaline-3-carboxylate (3d)**

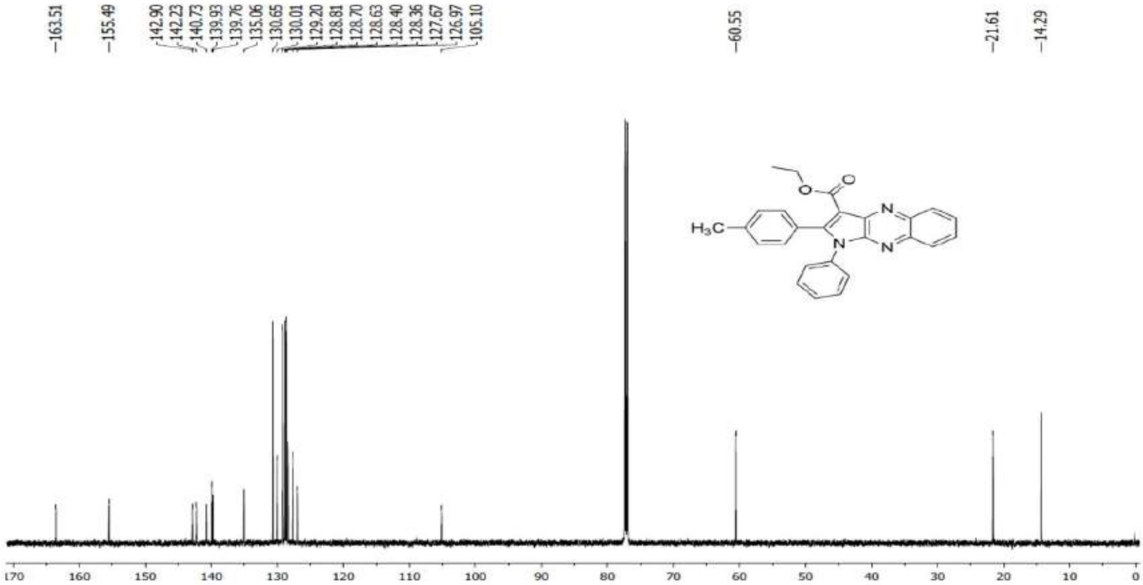

Figure S14. ^{13}C **NMR spectrum of ethyl 1-phenyl-2-(p-tolyl)-1H-pyrrolo[2,3-*b*]quinoxaline-3-carboxylate (3d)**

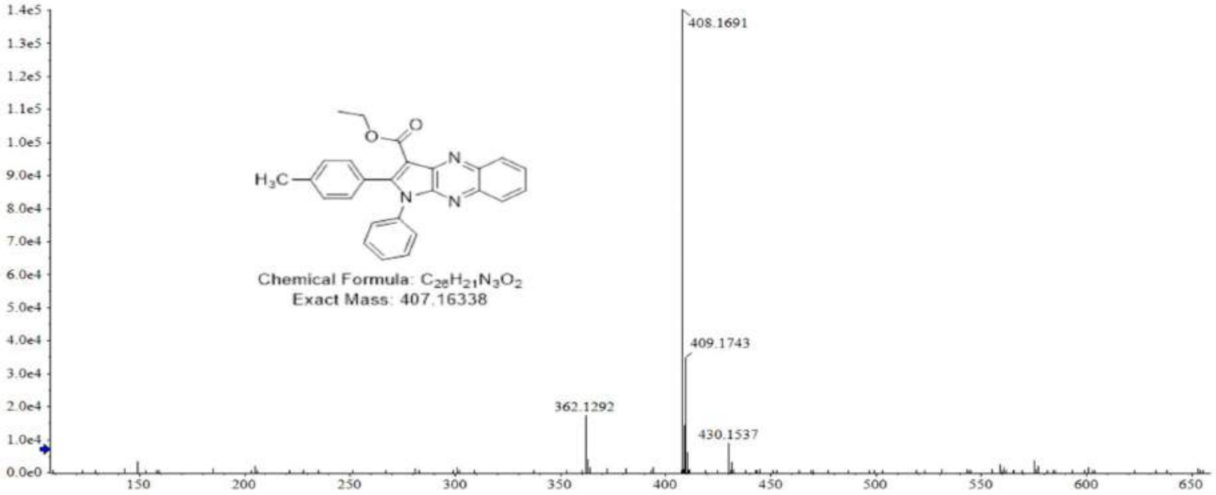

Figure S15. ESI-HRMS spectrum of ethyl 1-phenyl-2-(p-tolyl)-1H-pyrrolo[2,3-*b*]quinoxaline-3-carboxylate (3d)

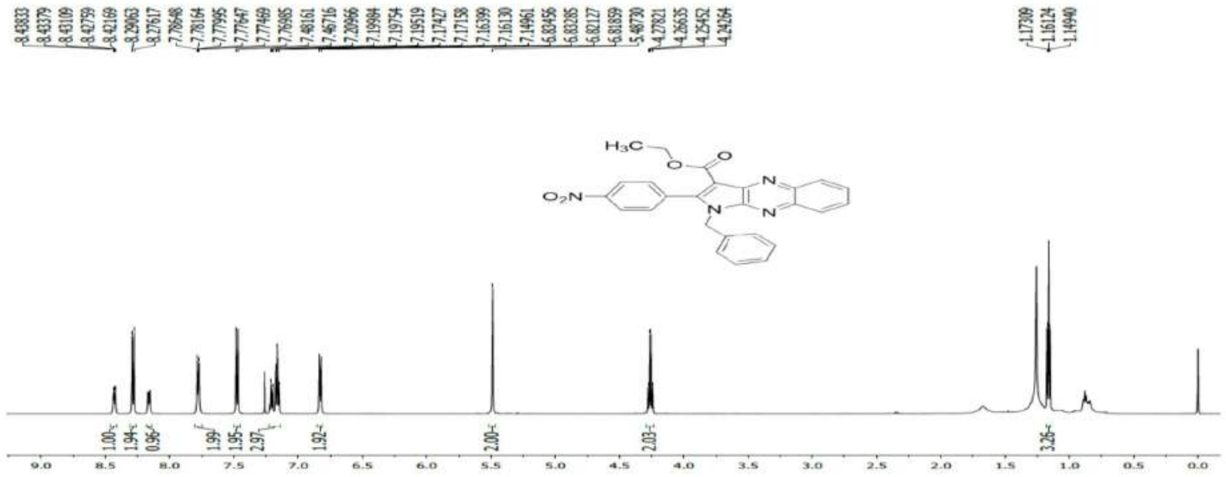

Figure S16. ^{1}H NMR spectrum of ethyl 1-benzyl-2-(4-nitrophenyl)-1H-pyrrolo[2,3-*b*]quinoxaline-3-carboxylate (3e)

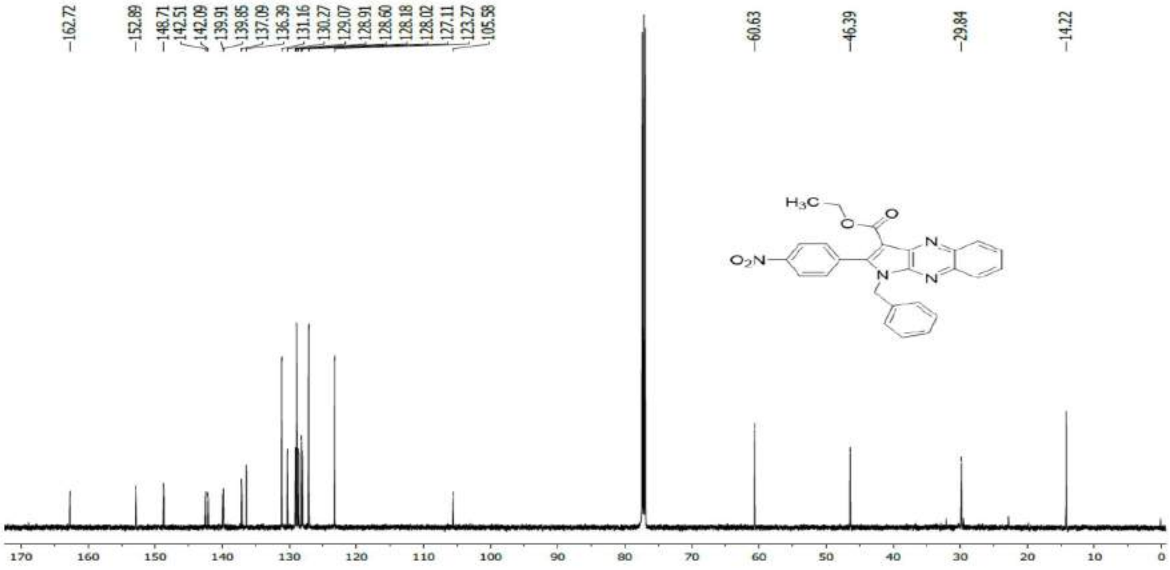

Figure S17. **^{13}C NMR spectrum of ethyl 1-benzyl-2-(4-nitrophenyl)-1H-pyrrolo[2,3-*b*]quinoxaline-3-carboxylate (3e)**

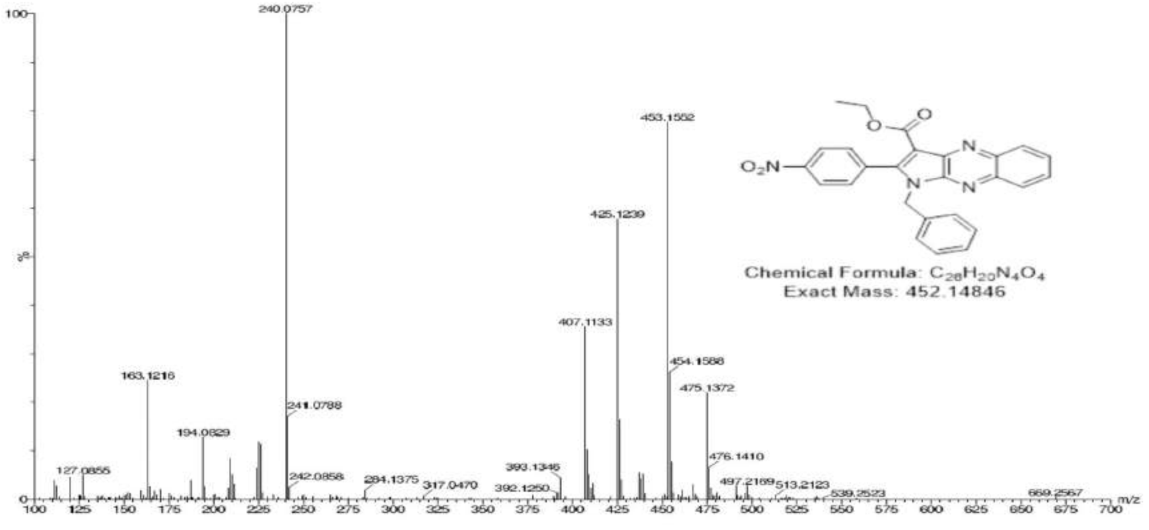

Figure S18. ESI-HRMS spectrum of ethyl 1-benzyl-2-(4-nitrophenyl)-1H-pyrrolo[2,3-*b*]quinoxaline-3-carboxylate (3e)

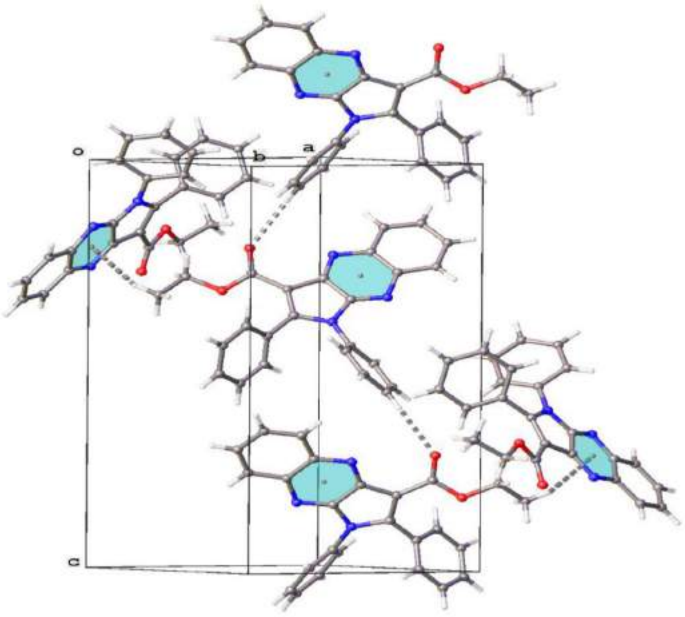

Figure S19. Partial crystal packing of X showing the chain formation along the *c* direction by C-H...O hydrogen bonding and C-H...π interactions.

Table S2. Crystal data and structure refinement details for 3a

Empirical formula	$C_{25}H_{19}N_3O_2$
Formula weight	393.43
Temperature/K	293(2)
Crystal system	orthorhombic
Space group	$P2_12_12_1$
a/Å	9.7669(5)
b/Å	10.7587(8)
c/Å	18.6574(14)
α/°	90
β/°	90
γ/°	90
Volume/Å^3	1960.5(2)
Z	4
ρ_{calc}g/cm^3	1.333
μ/mm^{-1}	0.086
F(000)	824.0
Crystal size/mm^3	0.5 × 0.3 × 0.15

Radiation	Mo Kα (λ = 0.71073 Å)
2Θ range for data collection/°	5.634 to 52.734
Index ranges	$-12 \leq h \leq 12$, $-12 \leq k \leq 13$, $-23 \leq l \leq 23$
Reflections collected	11625
Independent reflections	4001 [R_{int} = 0.0318, R_{sigma} = 0.0439]
Data/restraints/parameters	4001/0/272
Goodness-of-fit on F^2	1.073
Final R indexes [I>=2σ (I)]	R_1 = 0.0474, wR_2 = 0.0942
Final R indexes [all data]	R_1 = 0.0682, wR_2 = 0.1066
Largest diff. peak/hole / e $Å^{-3}$	0.19/-0.19
Flack parameter	-0.1(8)

Table S3: The Cartesian coordinates and energies of TS of the reaction between 4b with HO$^•$ and HOO$^•$ in the studied environments.

Name				TS-C2-OH-RAF-G
Cartesian Coordinates				Energy
C	1.26397900	-1.06735900	0.25955800	Zero-point correction= 0.399012 (Hartree/Particle)
C	1.44900800	0.36878800	0.09418200	Thermal correction to Energy= 0.424821
N	2.59252200	0.96313900	-0.00202400	Thermal correction to Enthalpy= 0.425765
C	3.65457100	0.10313400	0.00164300	Thermal correction to Gibbs Free Energy= 0.340756
C	3.48284500	-1.32509700	-0.02284800	Sum of electronic and zero-point Energies= -1353.977417
N	2.27492100	-1.91339500	0.03329500	Sum of electronic and thermal Energies= -1353.951608
C	-0.16830900	-1.29547900	0.13143400	Sum of electronic and thermal Enthalpies= -1353.950664
C	-0.76584000	-0.07266800	0.04706500	Sum of electronic and thermal Free Energies= -1354.035673
N	0.19941600	0.94305800	0.03090900	
C	-0.80467800	-2.60770400	0.30528900	
C	-2.20492800	0.24323500	0.01611100	
C	4.96203500	0.63632600	-0.04937400	
C	6.04879200	-0.19523900	-0.12839500	
C	5.88575200	-1.60436300	-0.17574000	
C	4.63691500	-2.15408400	-0.13094400	
C	-2.73372600	1.06966700	-0.97619800	
C	-4.09547300	1.34377700	-0.99924600	
C	-4.93324900	0.80509400	-0.02729500	
C	-4.40719400	-0.01212200	0.96925400	
C	-3.04739900	-0.29473700	0.99036100	
C	-0.03977500	2.34941500	0.07703900	
C	-0.84979700	2.88238200	1.07517400	
C	-1.08755600	4.25072500	1.10074800	
C	-0.50401900	5.08117600	0.14856800	
C	0.32068100	4.54061100	-0.83265000	
C	0.55280800	3.17080800	-0.87625800	
O	-0.36534100	-3.46506600	1.03079200	
O	-1.92909200	-2.73578000	-0.40750800	
C	-2.70337700	-3.91952900	-0.16253700	
C	-3.97053900	-3.78927500	-0.97708500	
H	5.06637200	1.71415100	-0.02506800	
H	7.04676000	0.22561800	-0.16122300	
H	6.75948000	-2.24083800	-0.24343000	
H	4.47527700	-3.22492000	-0.15555300	
H	-2.07865700	1.49231200	-1.72969700	
H	-4.50221300	1.98010300	-1.77580200	
H	-5.99400800	1.02549100	-0.04394200	
H	-5.05528600	-0.42581700	1.73268400	
H	-2.62750400	-0.92332100	1.76854100	
H	-1.28562600	2.22614500	1.81882600	
H	-1.72181600	4.66856000	1.87295600	
H	-0.68669800	6.14860600	0.17564200	
H	0.78330500	5.18493500	-1.57033900	
H	1.19400100	2.73445900	-1.63142700	
H	-2.11282400	-4.79295600	-0.44609400	
H	-2.90761900	-3.98716100	0.90900600	
H	-4.61231300	-4.65603600	-0.80991500	
H	-4.51408200	-2.88730400	-0.68982400	
H	-3.73742000	-3.72838900	-2.04090600	
O	1.37456900	-1.00346400	2.20105300	

H 1.19019500 -1.95029300 2.32769300	
**Name**	**TS-C4-OH-RAF-G**
Cartesian Coordinates	Energy
C 1.28230300 -1.10742000 0.18342200	Zero-point correction= 0.398543 (Hartree/Particle)
C 1.45679500 0.32333300 0.06330900	Thermal correction to Energy= 0.424542
N 2.59709700 0.94319000 -0.05930200	Thermal correction to Enthalpy= 0.425486
C 3.66781800 0.12612200 0.00719900	Thermal correction to Gibbs Free Energy= 0.339743
C 3.52444300 -1.29884700 0.29417700	Sum of electronic and zero-point Energies= -1353.971544
N 2.29260900 -1.92493600 0.22855700	Sum of electronic and thermal Energies= -1353.945544
C -0.14233600 -1.34258200 0.17563100	Sum of electronic and thermal Enthalpies= -1353.944600
C -0.75350800 -0.11558800 0.09372000	Sum of electronic and thermal Free Energies= -1354.030344
N 0.20277800 0.89855400 0.02205900	
C -0.78769300 -2.66757300 0.22390700	
C -2.19354700 0.19639700 0.09062600	
C 4.96556000 0.65649300 -0.16550100	
C 6.05092700 -0.17589300 -0.21625000	
C 5.90786100 -1.59075600 -0.10532800	
C 4.68763300 -2.14017400 0.11544400	
C -2.74360400 1.01411300 -0.89740100	
C -4.10646600 1.28436000 -0.89722200	
C -4.92451800 0.75213400 0.09456900	
C -4.37750500 -0.05725100 1.08656200	
C -3.01754200 -0.33771100 1.08306900	
C -0.03528900 2.30630000 0.05262900	
C -0.82417500 2.85727400 1.05782500	
C -1.05960600 4.22632300 1.06614400	
C -0.49544400 5.04008900 0.08853400	
C 0.30733300 4.48176600 -0.90093100	
C 0.53730700 3.11132400 -0.92648600	
O -0.33578500 -3.62839000 0.78647300	
O -1.95220900 -2.67588800 -0.44511600	
C -2.73250200 -3.87474700 -0.34568300	
C -4.03001400 -3.61168600 -1.07704900	
H 5.05937900 1.72891600 -0.28375600	
H 7.03896000 0.24266600 -0.36806500	
H 6.78161600 -2.22248500 -0.20580700	
H 4.53175600 -3.20902600 0.20015500	
H -2.10495200 1.43171000 -1.66751400	
H -4.52903800 1.91317700 -1.67143300	
H -5.98588700 0.97011800 0.09653000	
H -5.01009000 -0.46846100 1.86419900	
H -2.58442500 -0.96728800 1.85276500	
H -1.25056800 2.21549700 1.81934100	
H -1.67793000 4.65726800 1.84402000	
H -0.67634800 6.10804000 0.10168200	
H 0.75464000 5.11275000 -1.65926400	
H 1.16211000 2.66204900 -1.68767800	
H -2.16891100 -4.70224900 -0.78157000	
H -2.89408400 -4.10140000 0.71123600	
H -4.68059700 -4.48551600 -1.01112500	
H -4.54450700 -2.75442600 -0.63856800	
H -3.83935200 -3.39793600 -2.12963500	
O 3.62845700 -1.06089500 2.17044500	
H 3.24767600 -1.91628300 2.42367700	

Name				TS-C5-OH-RAF-G
Cartesian Coordinates				Energy
C	1.26808600	-1.08283100	0.10258100	Zero-point correction= 0.398546 (Hartree/Particle)
C	1.43935400	0.33737800	-0.02697500	Thermal correction to Energy= 0.424679
N	2.58131100	0.95426700	-0.09260600	Thermal correction to Enthalpy= 0.425624
C	3.65450100	0.12185300	-0.04091000	Thermal correction to Gibbs Free Energy= 0.338916
C	3.50374500	-1.29303400	0.05874100	Sum of electronic and zero-point Energies= -1353.980661
N	2.29955800	-1.89667900	0.13083400	Sum of electronic and thermal Energies= -1353.954527
C	-0.14754100	-1.32370000	0.14423900	Sum of electronic and thermal Enthalpies= -1353.953583
C	-0.76699800	-0.09495100	0.05783500	Sum of electronic and thermal Free Energies= -1354.040290
N	0.17908600	0.91595200	-0.04694500	
C	-0.78405900	-2.65271400	0.20762100	
C	-2.20849200	0.21023100	0.07520300	
C	4.95934300	0.67628500	-0.10742600	
C	6.06841400	-0.13700000	-0.09309800	
C	5.93436500	-1.53115200	-0.00275900	
C	4.67881700	-2.11432800	0.14231300	
C	-2.77545000	1.02136600	-0.90861300	
C	-4.13855700	1.28956200	-0.88842400	
C	-4.93984900	0.76172700	0.11926800	
C	-4.37578600	-0.04116200	1.10701300	
C	-3.01562400	-0.31954600	1.08359100	
C	-0.05240800	2.32399900	0.00256100	
C	-0.82573200	2.86999800	1.02255300	
C	-1.05045100	4.24070400	1.04847000	
C	-0.49048800	5.06127300	0.07419400	
C	0.29715600	4.50786400	-0.93010300	
C	0.51559600	3.13604900	-0.97340600	
O	-0.30960700	-3.61111400	0.75540000	
O	-1.96574600	-2.66837600	-0.42992500	
C	-2.73036900	-3.87662100	-0.31878200	
C	-4.04773100	-3.62446000	-1.01763700	
H	5.04203600	1.75431500	-0.17549900	
H	7.05682600	0.30125200	-0.15733200	
H	6.81406900	-2.16182000	0.00896300	
H	4.54420200	-3.18334800	0.04229700	
H	-2.14945600	1.43534200	-1.69103700	
H	-4.57445700	1.91318000	-1.65944000	
H	-6.00143200	0.97790000	0.13683400	
H	-4.99535500	-0.44905600	1.89678100	
H	-2.56965600	-0.94445700	1.84972000	
H	-1.24960200	2.22349000	1.78146800	
H	-1.65730900	4.66747900	1.83761000	
H	-0.66309900	6.13031000	0.10118300	
H	0.74095600	5.14374600	-1.68638900	
H	1.12728900	2.69074800	-1.74771100	
H	-2.16798600	-4.69510100	-0.77282600	
H	-2.86376000	-4.11104400	0.74031100	
H	-4.68630900	-4.50624900	-0.94139900	
H	-4.56182700	-2.77583800	-0.56229900	
H	-3.88463400	-3.40274000	-2.07319900	
O	4.70042200	-2.33906500	2.19368000	
H	3.80142700	-2.70491200	2.21531600	
Name				TS-C6-OH-RAF-G

Cartesian Coordinates	Energy
C 1.25888700 -1.11647000 0.11060600	Zero-point correction= 0.398558 (Hartree/Particle)
C 1.43643600 0.30427100 -0.01273800	Thermal correction to Energy= 0.424766
N 2.58464100 0.91668100 -0.08577300	Thermal correction to Enthalpy= 0.425710
C 3.65049500 0.08292400 -0.04529100	Thermal correction to Gibbs Free Energy= 0.338857
C 3.49675500 -1.33657700 0.05944800	Sum of electronic and zero-point Energies= -1353.979853
N 2.28134100 -1.93558000 0.13221000	Sum of electronic and thermal Energies= -1353.953645
C -0.16165800 -1.34868500 0.15052800	Sum of electronic and thermal Enthalpies= -1353.952701
C -0.77216600 -0.11820000 0.07095600	Sum of electronic and thermal Free Energies= -1354.039554
N 0.18383700 0.88942300 -0.02784500	
C -0.81124400 -2.67278400 0.20360900	
C -2.21149800 0.19693700 0.08775400	
C 4.96464600 0.63981900 -0.12671800	
C 6.05960200 -0.16209500 -0.09593100	
C 5.92603200 -1.58549100 0.05959100	
C 4.65545000 -2.15292900 0.06888700	
C -2.77263800 1.01566000 -0.89312100	
C -4.13450700 1.29025500 -0.87431900	
C -4.94059000 0.76063400 0.12862800	
C -4.38250800 -0.05026700 1.11319000	
C -3.02344800 -0.33434200 1.09152400	
C -0.04099500 2.29871000 0.02134600	
C -0.81462200 2.84710900 1.03985900	
C -1.03382200 4.21870900 1.06615400	
C -0.46854700 5.03752900 0.09346800	
C 0.31875500 4.48151600 -0.90965600	
C 0.53190300 3.10886900 -0.95334900	
O -0.35546600 -3.63596000 0.75757900	
O -1.98345900 -2.67434200 -0.45165800	
C -2.76551200 -3.87213700 -0.34918400	
C -4.07067900 -3.60260900 -1.06427400	
H 5.04286200 1.71662500 -0.21582100	
H 7.05445000 0.26201400 -0.14554200	
H 6.78812800 -2.21536600 -0.10746800	
H 4.51892000 -3.22508100 0.13152600	
H -2.14335000 1.43038600 -1.67250700	
H -4.56583200 1.91983200 -1.64306600	
H -6.00122700 0.98149800 0.14483300	
H -5.00578000 -0.45995600 1.89909900	
H -2.58205600 -0.96555300 1.85519800	
H -1.24363800 2.20172500 1.79685400	
H -1.64107000 4.64741500 1.85394000	
H -0.63712200 6.10720400 0.12053300	
H 0.76627600 5.11596800 -1.66493800	
H 1.14308200 2.66167600 -1.72693000	
H -2.20865700 -4.69857500 -0.79554900	
H -2.91513800 -4.10360300 0.70842100	
H -4.72243800 -4.47526600 -0.99482200	
H -4.57821200 -2.74627100 -0.61597200	
H -3.89174400 -3.38456800 -2.11803500	
O 6.32651400 -1.82663700 2.02708600	
H 5.53853600 -1.40482100 2.40260900	
**Name**	**TS-C7-OH-RAF-G**
Cartesian Coordinates	Energy

C	1.27724500	-1.11063900	0.10415500	Zero-point correction=	0.398516 (Hartree/Particle)
C	1.45448700	0.30973200	-0.02730500	Thermal correction to Energy=	0.424768
N	2.59825200	0.92060300	-0.10364100	Thermal correction to Enthalpy=	0.425712
C	3.66863800	0.08091300	-0.06151600	Thermal correction to Gibbs Free Energy=	0.338781
C	3.51228000	-1.33943300	0.03601500	Sum of electronic and zero-point Energies=	-1353.980371
N	2.30305600	-1.93282000	0.11884200	Sum of electronic and thermal Energies=	-1353.954119
C	-0.13866500	-1.34355000	0.15327000	Sum of electronic and thermal Enthalpies=	-1353.953175
C	-0.75276800	-0.11158700	0.07018000	Sum of electronic and thermal Free Energies=	-1354.040106
N	0.19747300	0.89487200	-0.03974800		
C	-0.78781200	-2.66763500	0.21399100		
C	-2.19298900	0.19752400	0.09407000		
C	4.96953500	0.62863700	-0.14750100		
C	6.09138200	-0.19144600	-0.06679700		
C	5.92109400	-1.61830900	-0.02545200		
C	4.68129100	-2.16624200	0.04334700		
C	-2.76120300	1.01837000	-0.88104700		
C	-4.12411500	1.28695500	-0.85592400		
C	-4.92407400	0.74973100	0.14788700		
C	-4.35888100	-0.06299100	1.12692500		
C	-2.99888200	-0.34164600	1.09864700		
C	-0.02608900	2.30415300	0.01513600		
C	-0.79264900	2.85069400	1.03996800		
C	-1.00927100	4.22256600	1.07182800		
C	-0.44794200	5.04349600	0.09863000		
C	0.33271100	4.48932400	-0.91064800		
C	0.54289000	3.11641500	-0.96005300		
O	-0.33493700	-3.62637300	0.77813100		
O	-1.95653700	-2.67466300	-0.44739800		
C	-2.74075900	-3.87030900	-0.33713600		
C	-4.04377200	-3.60445100	-1.05752200		
H	5.06782800	1.70349500	-0.23090500		
H	7.06462500	0.21543300	-0.30146800		
H	6.80338300	-2.24544400	-0.00898800		
H	4.52520300	-3.23650100	0.10337900		
H	-2.13654800	1.43919900	-1.66085400		
H	-4.56102200	1.91806300	-1.62023700		
H	-5.98551600	0.96623000	0.16920100		
H	-4.97737800	-0.47826000	1.91366500		
H	-2.55208400	-0.97399300	1.85826400		
H	-1.21784600	2.20380600	1.79784700		
H	-1.61074500	4.64993800	1.86475100		
H	-0.61388500	6.11344300	0.13045000		
H	0.77775800	5.12546900	-1.66594800		
H	1.14923700	2.67067700	-1.73825400		
H	-2.18414700	-4.70125200	-0.77537200		
H	-2.89339800	-4.09277600	0.72199400		
H	-4.69707900	-4.47551200	-0.98278500		
H	-4.55097100	-2.74383500	-0.61707600		
H	-3.86198700	-3.39495900	-2.11252100		
O	6.54631400	0.25162900	1.86382100		
H	5.73831800	-0.07959600	2.28499200		
**Name**				**TS-C8-OH-RAF-G**	
Cartesian Coordinates				Energy	
C	1.28284000	-1.07339600	0.11702500	Zero-point correction=	0.398555 (Hartree/Particle)

C	1.44659300	0.34783000	-0.01295500	Thermal correction to Energy= 0.424761
N	2.58798000	0.97092900	-0.08045600	Thermal correction to Enthalpy= 0.425705
C	3.65757300	0.14614100	-0.02965000	Thermal correction to Gibbs Free Energy= 0.338836
C	3.52041700	-1.27054400	0.05659000	Sum of electronic and zero-point Energies= -1353.981598
N	2.31464200	-1.88282000	0.13232300	Sum of electronic and thermal Energies= -1353.955392
C	-0.13448100	-1.31979700	0.15827600	Sum of electronic and thermal Enthalpies= -1353.954448
C	-0.75717100	-0.09464000	0.06947900	Sum of electronic and thermal Free Energies= -1354.041317
N	0.18713400	0.92051800	-0.03616100	
C	-0.76889900	-2.65026200	0.21468100	
C	-2.19959900	0.20733800	0.08409000	
C	4.97092600	0.72275400	-0.04726500	
C	6.08082600	-0.11190600	-0.14158400	
C	5.93721600	-1.51331300	-0.06538600	
C	4.69315900	-2.08095900	0.04107900	
C	-2.76818400	1.01000300	-0.90557100	
C	-4.13210100	1.27443800	-0.88811800	
C	-4.93261500	0.75108300	0.12251400	
C	-4.36688600	-0.04341600	1.11607800	
C	-3.00582900	-0.31774700	1.09554300	
C	-0.05107300	2.32815900	0.00491700	
C	-0.82907600	2.87563100	1.02033000	
C	-1.06021000	4.24530100	1.03828100	
C	-0.50219000	5.06309400	0.06059800	
C	0.29010200	4.50810400	-0.93902700	
C	0.51501800	3.13717400	-0.97440600	
O	-0.29450600	-3.61211100	0.75583600	
O	-1.95123000	-2.66229800	-0.42290600	
C	-2.71647800	-3.87021800	-0.31747500	
C	-4.03453800	-3.61283100	-1.01327800	
H	5.04494700	1.78446600	-0.23652200	
H	7.06632900	0.32931300	-0.22012600	
H	6.81857400	-2.14203900	-0.09674400	
H	4.55336500	-3.15339600	0.10405600	
H	-2.14276000	1.42086300	-1.69009500	
H	-4.56917800	1.89182300	-1.66348200	
H	-5.99482600	0.96435100	0.13790500	
H	-4.98582300	-0.44776800	1.90817500	
H	-2.55830700	-0.93620500	1.86599400	
H	-1.25047400	2.23138700	1.78248600	
H	-1.66980100	4.67368300	1.82441600	
H	-0.67900800	6.13156000	0.08202700	
H	0.73353400	5.14209300	-1.69707200	
H	1.13141500	2.69079300	-1.74429200	
H	-2.15561100	-4.68688100	-0.77673600	
H	-2.84891100	-4.11052300	0.74044700	
H	-4.67442000	-4.49400100	-0.94062500	
H	-4.54659400	-2.76558800	-0.55305500	
H	-3.87251300	-3.38598100	-2.06793000	
O	5.25469800	1.17651600	1.94550100	
H	5.38101500	0.27917800	2.29075300	
**Name**				**TS-C9-OH-RAF-G**
Cartesian Coordinates				Energy
C	1.26057300	-1.06970400	0.20999600	Zero-point correction= 0.398334 (Hartree/Particle)
C	1.43689400	0.35615500	0.04564500	Thermal correction to Energy= 0.424424

N 2.56774900 0.97254800 -0.04105200<br>C 3.65012400 0.13360600 0.15100200<br>C 3.48645000 -1.31706900 0.12604500<br>N 2.27994800 -1.90189600 0.19815000<br>C -0.15117400 -1.30581000 0.23602300<br>C -0.77195800 -0.07959000 0.10340100<br>N 0.17234900 0.92967500 -0.02289400<br>C -0.79647300 -2.63200800 0.30691900<br>C -2.21397500 0.21454700 0.08997900<br>C 4.95785500 0.67336700 -0.13832600<br>C 6.03411700 -0.14729800 -0.23432700<br>C 5.88021300 -1.55992500 -0.10204600<br>C 4.64828800 -2.12582300 0.06962800<br>C -2.76807900 1.02320500 -0.90376200<br>C -4.13448400 1.27368100 -0.91454900<br>C -4.95242400 0.73174800 0.07218700<br>C -4.40164600 -0.06790300 1.07015000<br>C -3.03817200 -0.32954900 1.07748400<br>C -0.05916700 2.33749100 0.01426600<br>C -0.84701600 2.89225000 1.01868400<br>C -1.06962000 4.26350800 1.03176200<br>C -0.49318900 5.07665600 0.06072900<br>C 0.30949000 4.51502500 -0.92695300<br>C 0.52601100 3.14245100 -0.95765500<br>O -0.36895000 -3.57096000 0.92068200<br>O -1.93211700 -2.66448400 -0.41064800<br>C -2.71816100 -3.85839000 -0.29820700<br>C -3.98795000 -3.61984900 -1.08452600<br>H 5.03311300 1.74940400 -0.24328700<br>H 7.01825100 0.26449700 -0.42112500<br>H 6.75802300 -2.19250700 -0.16072100<br>H 4.51104000 -3.19767700 0.14029600<br>H -2.12968100 1.44724100 -1.67050900<br>H -4.56021400 1.89440200 -1.69354200<br>H -6.01679700 0.93442400 0.06547300<br>H -5.03425300 -0.48625600 1.84390400<br>H -2.60248700 -0.95076800 1.85263200<br>H -1.28322300 2.25192700 1.77589600<br>H -1.68801600 4.69665400 1.80841700<br>H -0.66459600 6.14611400 0.07760100<br>H 0.76620100 5.14496800 -1.68057100<br>H 1.14940100 2.69037400 -1.71849100<br>H -2.14080100 -4.70107100 -0.68411700<br>H -2.91821300 -4.04785700 0.75951700<br>H -4.64209900 -4.49050600 -1.01218100<br>H -4.51640900 -2.74786000 -0.69420100<br>H -3.75933100 -3.44242200 -2.13630500<br>O 3.74274100 0.18915700 2.07402100<br>H 3.70974500 1.15289800 2.17354000	Thermal correction to Enthalpy= 0.425368<br>Thermal correction to Gibbs Free Energy= 0.339411<br>Sum of electronic and zero-point Energies= -1353.972014<br>Sum of electronic and thermal Energies= -1353.945924<br>Sum of electronic and thermal Enthalpies= -1353.944980<br>Sum of electronic and thermal Free Energies= -1354.030937
**Name**	**TS-C11-OH-RAF-G**
Cartesian Coordinates	Energy
C 1.26063300 -1.08519800 0.21733700<br>C 1.43562800 0.35996000 0.11179400<br>N 2.58944200 0.94531800 -0.18866500	Zero-point correction= 0.398425 (Hartree/Particle)<br>Thermal correction to Energy= 0.424403<br>Thermal correction to Enthalpy= 0.425348

C	3.64586000	0.11406100	-0.10175700	Thermal correction to Gibbs Free Energy= 0.339786
C	3.49244200	-1.30049600	0.12033400	Sum of electronic and zero-point Energies= -1353.977163
N	2.27867400	-1.90029600	0.22666100	Sum of electronic and thermal Energies= -1353.951184
C	-0.15133200	-1.31660800	0.21822400	Sum of electronic and thermal Enthalpies= -1353.950240
C	-0.76299500	-0.09108900	0.03257300	Sum of electronic and thermal Free Energies= -1354.035802
N	0.17267600	0.91276700	-0.12753800	
C	-0.79329100	-2.64181500	0.30425000	
C	-2.20225600	0.21697400	-0.00101700	
C	4.95434300	0.64876600	-0.28131300	
C	6.04784100	-0.16499600	-0.22041900	
C	5.89574400	-1.55978900	0.00921400	
C	4.65422000	-2.11180400	0.16637400	
C	-2.73460500	1.00593300	-1.02139200	
C	-4.09577400	1.28329000	-1.04652700	
C	-4.92727100	0.79051300	-0.04572100	
C	-4.39616800	0.01359100	0.98070300	
C	-3.03895300	-0.27730500	1.00134600	
C	-0.06756000	2.31555400	0.00363100	
C	-0.75751600	2.80501800	1.10882200	
C	-0.98434700	4.17131400	1.21697400	
C	-0.50531500	5.04034500	0.24041800	
C	0.20451600	4.54271500	-0.84751000	
C	0.42366300	3.17513300	-0.97192300	
O	-0.34200500	-3.58510900	0.89430200	
O	-1.95442400	-2.67350300	-0.37284100	
C	-2.72475400	-3.87632000	-0.25147800	
C	-4.01719600	-3.64419000	-1.00217600	
H	5.03800400	1.71509900	-0.45364800	
H	7.04086700	0.24864800	-0.34783900	
H	6.77634000	-2.18939300	0.05334900	
H	4.51170800	-3.17248600	0.33265000	
H	-2.08349400	1.39231000	-1.79722000	
H	-4.50624900	1.88757700	-1.84634300	
H	-5.98720800	1.01486400	-0.06279500	
H	-5.03971900	-0.36429400	1.76618300	
H	-2.61665300	-0.87956500	1.79828100	
H	-1.10116500	2.11428400	1.86926400	
H	-1.52778600	4.55812400	2.07043700	
H	-0.68092800	6.10558500	0.33083700	
H	0.58543400	5.21790100	-1.60406800	
H	0.97475800	2.76664300	-1.80986400	
H	-2.14936000	-4.70919400	-0.66103100	
H	-2.89500400	-4.07872700	0.80897300	
H	-4.65945600	-4.52302000	-0.92215200	
H	-4.54535100	-2.78234600	-0.58978900	
H	-3.81776500	-3.45338200	-2.05757300	
O	1.48397500	0.60778800	2.05799100	
H	2.03637600	1.40505200	2.08589000	
**Name**				**TS-C12-OH-RAF-G**
Cartesian Coordinates				Energy
C	1.25304200	-1.03772200	0.28582700	Zero-point correction= 0.399336 (Hartree/Particle)
C	1.42106700	0.35886800	0.00290400	Thermal correction to Energy= 0.425136
N	2.55236600	0.95647400	-0.17981800	Thermal correction to Enthalpy= 0.426080
C	3.63075100	0.12072400	-0.09808100	Thermal correction to Gibbs Free Energy= 0.341050

C	3.48204900	-1.27702700	0.15242900	Sum of electronic and zero-point Energies= -1353.989730
N	2.26332400	-1.85591300	0.34984100	Sum of electronic and thermal Energies= -1353.963930
C	-0.17248800	-1.25538600	0.47103800	Sum of electronic and thermal Enthalpies= -1353.962986
C	-0.79333600	-0.02708600	0.18580000	Sum of electronic and thermal Free Energies= -1354.048016
N	0.15330400	0.94295100	-0.03566200	
C	-0.80690400	-2.59885600	0.38815700	
C	-2.22965900	0.25846000	0.19268300	
C	4.92818200	0.65703800	-0.28002600	
C	6.02631300	-0.15863800	-0.21785900	
C	5.88094200	-1.54644000	0.02674200	
C	4.63940100	-2.09328300	0.20775400	
C	-2.81018400	1.05360600	-0.79941300	
C	-4.18020000	1.27776900	-0.79312700	
C	-4.97352400	0.72476500	0.20865700	
C	-4.39437800	-0.05699400	1.20477200	
C	-3.02652400	-0.29690500	1.19912000	
C	-0.06030300	2.35588100	-0.04693900	
C	-0.76192200	2.94950400	0.99747500	
C	-0.97426400	4.32187900	0.97556100	
C	-0.47420900	5.09163700	-0.07095200	
C	0.24366800	4.48812600	-1.09848100	
C	0.45213600	3.11378100	-1.09328800	
O	-0.39652500	-3.57289000	0.95807400	
O	-1.87575800	-2.59290300	-0.41480300	
C	-2.64421100	-3.80549900	-0.45820200	
C	-3.87188600	-3.51215500	-1.29056200	
H	5.01198000	1.72107300	-0.46513900	
H	7.01695500	0.25748100	-0.35642300	
H	6.76188400	-2.17517500	0.07243000	
H	4.49640700	-3.14966500	0.40010300	
H	-2.19148700	1.48219600	-1.57965500	
H	-4.62899000	1.88501400	-1.56981800	
H	-6.04168200	0.90743400	0.21490400	
H	-5.00942800	-0.47730500	1.99158300	
H	-2.55058000	-0.88527200	1.97635100	
H	-1.13081000	2.33517300	1.81077600	
H	-1.52322700	4.79068300	1.78296600	
H	-0.63805100	6.16240100	-0.08150300	
H	0.64132500	5.08643900	-1.90909900	
H	1.01096800	2.62730000	-1.88272000	
H	-2.02745700	-4.59833000	-0.88634300	
H	-2.89759100	-4.09175500	0.56511600	
H	-4.51260500	-4.39455600	-1.33405200	
H	-4.43667800	-2.68720900	-0.85215800	
H	-3.58950000	-3.23652000	-2.30754200	
O	-0.24041500	-1.07771600	2.50565300	
H	0.21636900	-1.92228600	2.64587500	
**Name**				**TS-C12-OH-RAF-P**
Cartesian Coordinates				Energy
C	1.23796200	-1.03737800	0.30704400	Zero-point correction= 0.399028 (Hartree/Particle)
C	1.40911000	0.36053000	0.03033600	Thermal correction to Energy= 0.424823
N	2.54334900	0.95364000	-0.14744000	Thermal correction to Enthalpy= 0.425767
C	3.62209100	0.11659500	-0.07350500	Thermal correction to Gibbs Free Energy= 0.340877
C	3.47078700	-1.28254500	0.16876900	Sum of electronic and zero-point Energies= -1354.023273

N	2.24853100	-1.85669100	0.36595500	Sum of electronic and thermal Energies= -1353.997478
C	-0.18640900	-1.25071200	0.49714400	Sum of electronic and thermal Enthalpies= -1353.996534
C	-0.80429100	-0.01440000	0.22217300	Sum of electronic and thermal Free Energies= -1354.081424
N	0.14524800	0.94850300	0.00004200	
C	-0.82018700	-2.59336100	0.39773600	
C	-2.23913900	0.27560500	0.23726300	
C	4.91994500	0.65248300	-0.25328700	
C	6.01705300	-0.16637100	-0.19718400	
C	5.86908000	-1.55538100	0.03878500	
C	4.62615000	-2.10181200	0.21793300	
C	-2.81973500	1.10365700	-0.72943100	
C	-4.18936000	1.33217500	-0.71128600	
C	-4.98251600	0.75175600	0.27578000	
C	-4.40490900	-0.06672600	1.24350900	
C	-3.03813800	-0.31246200	1.22423600	
C	-0.06128700	2.36514800	-0.04590500	
C	-0.69433100	2.99637900	1.01949300	
C	-0.90310500	4.36928200	0.96288600	
C	-0.46805300	5.09863600	-0.14060000	
C	0.17932600	4.45561700	-1.19189700	
C	0.38352200	3.08062700	-1.15147300	
O	-0.40842100	-3.56935700	0.97137000	
O	-1.86717000	-2.59338600	-0.42401600	
C	-2.62618600	-3.81711700	-0.50259400	
C	-3.81200600	-3.54433300	-1.39592800	
H	5.00978000	1.71763400	-0.43320700	
H	7.00879800	0.24845000	-0.33412500	
H	6.74925300	-2.18618000	0.07920400	
H	4.48724300	-3.16062600	0.40386300	
H	-2.20582900	1.55079500	-1.50294600	
H	-4.63789400	1.96429100	-1.46852100	
H	-6.05007300	0.93924500	0.29111400	
H	-5.01979800	-0.51292100	2.01639300	
H	-2.56992600	-0.93446800	1.97931700	
H	-1.01699700	2.41421100	1.87534700	
H	-1.39956000	4.86892700	1.78612300	
H	-0.62996800	6.16949400	-0.17882800	
H	0.52240600	5.02230300	-2.04929700	
H	0.87683500	2.56391800	-1.96597000	
H	-1.98248100	-4.60393900	-0.90042300	
H	-2.93135400	-4.09872600	0.50767300	
H	-4.42874400	-4.44246500	-1.46732200	
H	-4.42115000	-2.73431900	-0.98921100	
H	-3.48569900	-3.26800900	-2.40026000	
O	-0.22642400	-1.10961000	2.55112500	
H	0.22232400	-1.96343400	2.65496800	
**Name**				**TS-C13-OH-RAF-G**
Cartesian Coordinates				Energy
C	1.27109400	-1.06420100	0.10735900	Zero-point correction= 0.398778 (Hartree/Particle)
C	1.42840600	0.36036200	-0.02901100	Thermal correction to Energy= 0.424724
N	2.56008100	0.97672600	-0.15722000	Thermal correction to Enthalpy= 0.425668
C	3.64143300	0.14378400	-0.17524000	Thermal correction to Gibbs Free Energy= 0.339050
C	3.50348600	-1.27591700	-0.06816700	Sum of electronic and zero-point Energies= -1353.987608
N	2.29667300	-1.88012400	0.07745800	Sum of electronic and thermal Energies= -1353.961661

C	-0.13605500	-1.31107500	0.23388200	Sum of electronic and thermal Enthalpies=	-1353.960717
C	-0.77565300	-0.04951800	0.22794600	Sum of electronic and thermal Free Energies=	-1354.047335
N	0.16514400	0.93917700	0.00937800		
C	-0.76240500	-2.64206200	0.36876600		
C	-2.21178300	0.25971700	0.01464400		
C	4.93479600	0.70325800	-0.31066300		
C	6.03726700	-0.10815700	-0.34203000		
C	5.90380100	-1.51627000	-0.24030200		
C	4.66858900	-2.08688900	-0.10627700		
C	-2.55173800	0.96066200	-1.14436600		
C	-3.88289200	1.23994800	-1.42791900		
C	-4.88058700	0.83125700	-0.55021100		
C	-4.54131200	0.13768800	0.60797600		
C	-3.21345900	-0.15449900	0.89199900		
C	-0.06282500	2.34283300	0.15690200		
C	-0.82659300	2.80586300	1.22382300		
C	-1.05720100	4.17099900	1.34420000		
C	-0.51590400	5.06066200	0.42134000		
C	0.26085600	4.58414300	-0.63019700		
C	0.48746600	3.22029000	-0.77095600		
O	-0.22171100	-3.58386500	0.88130500		
O	-1.99323600	-2.67113800	-0.15315900		
C	-2.71822800	-3.90038800	0.00934700		
C	-4.07624600	-3.69555500	-0.62283900		
H	5.01188400	1.78112600	-0.38553200		
H	7.02420400	0.32680000	-0.44504700		
H	6.78955900	-2.13916100	-0.26710200		
H	4.53194900	-3.15816000	-0.02213100		
H	-1.77343100	1.28370800	-1.82641700		
H	-4.13717800	1.78084400	-2.33129200		
H	-5.91859900	1.05604500	-0.76532900		
H	-5.31564900	-0.17587300	1.29835300		
H	-2.93877800	-0.68598700	1.79238700		
H	-1.21952600	2.09523100	1.94122200		
H	-1.65559300	4.53959500	2.16847700		
H	-0.69571500	6.12410200	0.52321300		
H	0.68842300	5.27396400	-1.34763900		
H	1.08922400	2.83366100	-1.58359900		
H	-2.15504600	-4.70593000	-0.46623900		
H	-2.78723300	-4.12408700	1.07641900		
H	-4.67828500	-4.59898900	-0.51100300		
H	-4.59666700	-2.86321800	-0.14657400		
H	-3.97409700	-3.47390600	-1.68608100		
O	-0.79105500	-0.28304800	2.31548400		
H	0.05507400	-0.68625800	2.56238300		
**Name**				**TS-C13-OH-RAF-P**	
Cartesian Coordinates				Energy	
C	1.29695000	-1.09096600	0.02315100	Zero-point correction=	0.398562 (Hartree/Particle)
C	1.44217700	0.34056100	-0.03131100	Thermal correction to Energy=	0.424311
N	2.56822100	0.97653000	-0.08710100	Thermal correction to Enthalpy=	0.425255
C	3.66492200	0.16343400	-0.11742200	Thermal correction to Gibbs Free Energy=	0.341285
C	3.54398500	-1.26061700	-0.08287500	Sum of electronic and zero-point Energies=	-1354.019809
N	2.33947800	-1.88519300	-0.00391200	Sum of electronic and thermal Energies=	-1353.994060

C	-0.11423200	-1.35822500	0.10350300	Sum of electronic and thermal Enthalpies=	-1353.993116
C	-0.76542300	-0.09819900	0.13518100	Sum of electronic and thermal Free Energies=	-1354.077086
N	0.17285600	0.90320000	0.00667900		
C	-0.72585500	-2.69925900	0.10656100		
C	-2.19067900	0.23831600	-0.11789800		
C	4.95230700	0.74792200	-0.18570600		
C	6.06773800	-0.04673900	-0.22188600		
C	5.95183600	-1.45961900	-0.19084200		
C	4.72113000	-2.05344600	-0.12230700		
C	-2.47265500	0.92214200	-1.30360500		
C	-3.78390800	1.25596800	-1.62295600		
C	-4.81692200	0.92205300	-0.75355400		
C	-4.53418900	0.24552900	0.43037600		
C	-3.22747100	-0.10310900	0.74917100		
C	-0.07798900	2.30218900	0.17183700		
C	-0.84899900	2.73457800	1.24642800		
C	-1.10114200	4.09396800	1.39233300		
C	-0.57388300	5.00721300	0.48344600		
C	0.20622700	4.56066500	-0.57965800		
C	0.45420700	3.20244700	-0.74473200		
O	-0.08921500	-3.71902100	0.19982000		
O	-2.05149800	-2.65708700	-0.01621100		
C	-2.74005400	-3.92195000	0.00221900		
C	-4.21943900	-3.62280400	-0.04459200		
H	5.01935000	1.82953300	-0.20766700		
H	7.05124100	0.40539700	-0.27423100		
H	6.84759200	-2.06858200	-0.21995500		
H	4.60386200	-3.13065100	-0.09493300		
H	-1.66689900	1.18675800	-1.97988200		
H	-3.99413600	1.78049000	-2.54760900		
H	-5.83913000	1.18893600	-0.99638200		
H	-5.33680600	-0.01517500	1.11077500		
H	-3.00272500	-0.63307100	1.66345800		
H	-1.23960700	2.00705200	1.94878100		
H	-1.70576400	4.43875700	2.22284300		
H	-0.77020200	6.06624100	0.60361200		
H	0.61796400	5.26901100	-1.28882000		
H	1.04974300	2.84105700	-1.57424500		
H	-2.41390600	-4.50876200	-0.85933200		
H	-2.46059600	-4.46016200	0.91006900		
H	-4.77890700	-4.56034900	-0.06805000		
H	-4.52806700	-3.05755000	0.83670400		
H	-4.47044900	-3.04537700	-0.93668100		
O	-0.83820900	-0.52020200	2.21367600		
H	0.09189200	-0.65566100	2.45545800		
**Name**				**TS-C14-OH-RAF-G**	
Cartesian Coordinates				Energy	
C	1.33459900	-1.08827800	0.17771800	Zero-point correction=	0.398991 (Hartree/Particle)
C	1.52864500	0.33669200	0.14785400	Thermal correction to Energy=	0.424632
N	2.68296900	0.92844800	0.05527200	Thermal correction to Enthalpy=	0.425577
C	3.74310100	0.07505400	-0.00048100	Thermal correction to Gibbs Free Energy=	0.341280
C	3.56555100	-1.34227900	0.02464100	Sum of electronic and zero-point Energies=	-1353.973791
N	2.34031700	-1.92126300	0.10864300	Sum of electronic and thermal Energies=	-1353.948149
C	-0.08937700	-1.30443200	0.22131400	Sum of electronic and thermal Enthalpies=	-1353.947205

C	-0.68367700	-0.06529000	0.22628400	Sum of electronic and thermal Free Energies= -1354.031502
N	0.28327500	0.93996100	0.21471900	
C	-0.75084200	-2.62258800	0.17818700	
C	-2.12608800	0.24548100	0.19862800	
C	5.05362000	0.60847500	-0.09183200	
C	6.13435600	-0.22743200	-0.15383300	
C	5.96189300	-1.63559000	-0.12934000	
C	4.71091700	-2.17941700	-0.04290100	
C	-2.66016600	0.99728600	-0.84797900	
C	-4.02243600	1.26806800	-0.88415300	
C	-4.85394600	0.80033800	0.12877900	
C	-4.32184100	0.05282300	1.17621600	
C	-2.96225700	-0.22962400	1.20880500	
C	0.06357100	2.35647800	0.26064000	
C	-1.03432100	2.87326300	1.00579100	
C	-1.46740400	4.15890800	0.78146500	
C	-0.88437300	4.94118400	-0.22087400	
C	0.13893600	4.42035400	-1.01297100	
C	0.58829300	3.13030000	-0.81065700	
O	-0.27679700	-3.63797500	0.61008500	
O	-1.95462700	-2.55629000	-0.41444600	
C	-2.73162200	-3.76127400	-0.40917800	
C	-4.06425800	-3.42194600	-1.03908000	
H	5.16141900	1.68647200	-0.10734500	
H	7.13361100	0.18583800	-0.22055600	
H	6.83147400	-2.27969300	-0.17791500	
H	4.54585000	-3.24973400	-0.02001700	
H	-2.00757100	1.36739100	-1.63162500	
H	-4.43354400	1.84753800	-1.70186900	
H	-5.91502800	1.01827700	0.10281200	
H	-4.96641600	-0.31065500	1.96769700	
H	-2.53990900	-0.81406000	2.01883100	
H	-1.45837700	2.26742000	1.79492600	
H	-2.26741500	4.56393200	1.38838500	
H	-1.23623200	5.95171100	-0.38909500	
H	0.57010100	5.01868100	-1.80614100	
H	1.35855000	2.69580300	-1.43560800	
H	-2.19331300	-4.53246100	-0.96417700	
H	-2.83864400	-4.10602100	0.62219700	
H	-4.71240100	-4.30008400	-1.03990500	
H	-4.55449900	-2.62183400	-0.48115000	
H	-3.92694300	-3.08915700	-2.06889700	
O	1.33280900	2.85342900	1.67177900	
H	2.14973800	2.67247000	1.17701900	
**Name**				**TS-C15-OH-RAF-G**
Cartesian Coordinates				Energy
C	1.24345400	-1.05787900	0.10660700	Zero-point correction= 0.398750 (Hartree/Particle)
C	1.39969900	0.36209300	-0.05692700	Thermal correction to Energy= 0.424864
N	2.53033400	0.99485900	-0.11338000	Thermal correction to Enthalpy= 0.425808
C	3.61792200	0.17817500	-0.02603600	Thermal correction to Gibbs Free Energy= 0.339099
C	3.48524200	-1.23950900	0.10140000	Sum of electronic and zero-point Energies= -1353.981269
N	2.27674700	-1.85670900	0.16743500	Sum of electronic and thermal Energies= -1353.955156
C	-0.17649300	-1.31233200	0.14206600	Sum of electronic and thermal Enthalpies= -1353.954212
C	-0.80640800	-0.09596100	0.02661600	Sum of electronic and thermal Free Energies= -1354.040921

N	0.13093300	0.92399300	-0.11402700	
C	-0.80042100	-2.64671000	0.21864900	
C	-2.24638000	0.21334600	0.03322200	
C	4.91402500	0.75095300	-0.07254500	
C	6.02332900	-0.04605300	0.00008900	
C	5.89505100	-1.45366700	0.12207900	
C	4.65915500	-2.03557200	0.17189400	
C	-2.80125300	1.02206300	-0.95970800	
C	-4.16038800	1.31246500	-0.94185300	
C	-4.96867300	0.80839000	0.07206800	
C	-4.41609300	0.00784600	1.06866100	
C	-3.06063500	-0.29172000	1.04881100	
C	-0.09864100	2.31937900	-0.02882700	
C	-0.82992800	2.84148400	1.06694300	
C	-1.06216900	4.23535500	1.10935000	
C	-0.47213500	5.07319800	0.18421100	
C	0.31836900	4.54055700	-0.83959000	
C	0.50346700	3.16509500	-0.94798200	
O	-0.30765900	-3.60256600	0.75293100	
O	-1.99693300	-2.67104000	-0.39355500	
C	-2.74205900	-3.89046000	-0.27633200	
C	-4.07510700	-3.65404800	-0.95066200	
H	4.98603200	1.82787700	-0.16624400	
H	7.01103200	0.39786500	-0.03501700	
H	6.78617900	-2.06699100	0.17870700	
H	4.52782800	-3.10635000	0.26973900	
H	-2.17013400	1.41336200	-1.74977000	
H	-4.58735800	1.93312200	-1.72026500	
H	-6.02671000	1.04137100	0.08818700	
H	-5.04123200	-0.37992200	1.86413400	
H	-2.62181800	-0.90941100	1.82451400	
H	-1.43256600	2.17744600	1.67034400	
H	-1.67375600	4.63514700	1.90791300	
H	-0.62406100	6.14371100	0.24655500	
H	0.78394100	5.19898400	-1.56270100	
H	1.11258800	2.74397800	-1.73719200	
H	-2.17622800	-4.69808100	-0.74540400	
H	-2.85336600	-4.13379400	0.78325200	
H	-4.69847100	-4.54628000	-0.86990700	
H	-4.59495500	-2.81700300	-0.48078500	
H	-3.93376000	-3.42233400	-2.00724200	
O	0.59843700	2.58879300	2.43358600	
H	1.37476400	2.89759700	1.93892700	
**Name**				**TS-C16-OH-RAF-G**
Cartesian Coordinates				Energy
C	1.26441900	-1.10587600	0.10531900	Zero-point correction= 0.398539 (Hartree/Particle)
C	1.43215500	0.31773400	-0.01420800	Thermal correction to Energy= 0.424689
N	2.56926100	0.94089500	-0.07287100	Thermal correction to Enthalpy= 0.425633
C	3.64894000	0.11178500	-0.02555600	Thermal correction to Gibbs Free Energy= 0.338707
C	3.50436500	-1.30810300	0.06590300	Sum of electronic and zero-point Energies= -1353.977961
N	2.29165500	-1.91578800	0.12978600	Sum of electronic and thermal Energies= -1353.951811
C	-0.15646700	-1.34820900	0.13969700	Sum of electronic and thermal Enthalpies= -1353.950867
C	-0.77625300	-0.12259300	0.06021600	Sum of electronic and thermal Free Energies= -1354.037793
N	0.17045900	0.89245300	-0.03794100	

C	-0.79637500	-2.67657900	0.18570200	
C	-2.21700100	0.18715000	0.07698600	
C	4.95026000	0.67339200	-0.07757800	
C	6.05232100	-0.13557300	-0.04302400	
C	5.91216400	-1.54510000	0.04408100	
C	4.67195300	-2.11668400	0.09748200	
C	-2.78120400	0.99642000	-0.90999300	
C	-4.14289500	1.27237800	-0.88932100	
C	-4.94544300	0.75367200	0.12218600	
C	-4.38410500	-0.04749200	1.11283000	
C	-3.02536800	-0.33316100	1.08911200	
C	-0.05755800	2.29823400	0.02725200	
C	-0.82750100	2.83754300	1.04115700	
C	-1.00544700	4.23886600	1.11764200	
C	-0.48259200	5.05216500	0.08308800	
C	0.29670200	4.49473700	-0.91383800	
C	0.51608200	3.11743400	-0.94936800	
O	-0.32424000	-3.64582000	0.71485800	
O	-1.98223200	-2.67645600	-0.44624900	
C	-2.75064900	-3.88338200	-0.35189800	
C	-4.07088600	-3.61406900	-1.03898900	
H	5.03091000	1.75174100	-0.14323400	
H	7.04372100	0.29970500	-0.08166800	
H	6.79810400	-2.16790600	0.07052400	
H	4.53189800	-3.18849200	0.16822200	
H	-2.15433100	1.40123100	-1.69667700	
H	-4.57699300	1.89344600	-1.66346300	
H	-6.00583400	0.97548500	0.14025700	
H	-5.00465600	-0.44816800	1.90548000	
H	-2.58126600	-0.95658700	1.85745100	
H	-1.25974100	2.19859700	1.80082400	
H	-1.76659800	4.63538200	1.77456800	
H	-0.65904200	6.11965500	0.11488500	
H	0.73275600	5.12352100	-1.68010800	
H	1.12631600	2.66977100	-1.72336100	
H	-2.19407600	-4.69597900	-0.82341100	
H	-2.87921700	-4.13614400	0.70356100	
H	-4.71277900	-4.49439100	-0.97443000	
H	-4.57905700	-2.77131300	-0.56636300	
H	-3.91270000	-3.37482500	-2.09149400	
O	0.28480700	4.65377900	2.56299800	
H	1.09141300	4.29114500	2.16450300	
**Name**				**TS-C18-OH-RAF-G**
Cartesian Coordinates				Energy
C	1.23217600	-1.03305500	0.08144400	Zero-point correction= 0.398857 (Hartree/Particle)
C	1.36866200	0.39034400	-0.06519600	Thermal correction to Energy= 0.424858
N	2.49083200	1.04363300	-0.10645100	Thermal correction to Enthalpy= 0.425802
C	3.59033500	0.24278200	-0.03060400	Thermal correction to Gibbs Free Energy= 0.339211
C	3.47754100	-1.17941700	0.07792300	Sum of electronic and zero-point Energies= -1353.978784
N	2.27928300	-1.81611800	0.13623200	Sum of electronic and thermal Energies= -1353.952784
C	-0.18478100	-1.30424300	0.11492900	Sum of electronic and thermal Enthalpies= -1353.951839
C	-0.83035100	-0.09262100	0.00860100	Sum of electronic and thermal Free Energies= -1354.038431
N	0.09601200	0.93518900	-0.11875300	
C	-0.79481000	-2.64482900	0.18975000	

C -2.27237800 0.20522800 0.03670300<br>C 4.87751800 0.83635600 -0.07012200<br>C 5.99793300 0.05485600 -0.00760900<br>C 5.89056800 -1.35639600 0.09706200<br>C 4.66420300 -1.95801200 0.13935300<br>C -2.84644700 1.02691300 -0.93446100<br>C -4.20872400 1.29897700 -0.89953800<br>C -5.00195400 0.76438900 0.11065600<br>C -4.43083200 -0.04825200 1.08652600<br>C -3.07188500 -0.32980900 1.04888200<br>C -0.11875200 2.34229500 -0.05184200<br>C -0.79281600 2.90884200 1.02970700<br>C -0.90447400 4.29927900 1.11751600<br>C -0.30162600 5.10845900 0.17693300<br>C 0.45247100 4.53922200 -0.88250900<br>C 0.43597200 3.13358500 -1.04159900<br>O -0.29532000 -3.59466500 0.72911800<br>O -1.98741600 -2.68207400 -0.42874200<br>C -2.72215200 -3.90807000 -0.31258600<br>C -4.05440100 -3.68477500 -0.99283200<br>H 4.93415200 1.91535100 -0.15392400<br>H 6.97881800 0.51376200 -0.03893800<br>H 6.79099700 -1.95667200 0.14559200<br>H 4.54954400 -3.03189400 0.22288000<br>H -2.22740300 1.44291600 -1.72122900<br>H -4.65023300 1.92967800 -1.66154400<br>H -6.06278500 0.98289200 0.13974200<br>H -5.04435700 -0.46082800 1.87856100<br>H -2.62045900 -0.96154800 1.80596500<br>H -1.22156300 2.26630500 1.78959800<br>H -1.44995800 4.73773500 1.94390200<br>H -0.35762200 6.18612700 0.26190800<br>H 0.73922000 5.15904600 -1.72009400<br>H 0.94834400 2.67458000 -1.87781500<br>H -2.14699100 -4.71126400 -0.77782000<br>H -2.83574500 -4.15053200 0.74700000<br>H -4.66993200 -4.58252000 -0.91332400<br>H -4.58382600 -2.85182700 -0.52635800<br>H -3.91076000 -3.45344400 -2.04915500<br>O 2.28102300 4.66417200 -0.20270700<br>H 2.42936200 3.74234400 0.07030400	
**Name**	**TS-C19-OH-RAF-G**
Cartesian Coordinates	Energy
C 1.29814900 -1.06286700 0.18235100<br>C 1.49940700 0.34240400 -0.04380100<br>N 2.64718000 0.89194000 -0.28379800<br>C 3.69603200 0.02613900 -0.26011800<br>C 3.51477600 -1.37157200 -0.02877300<br>N 2.28828700 -1.91660200 0.17758000<br>C -0.12337500 -1.26141500 0.29536800<br>C -0.71719700 -0.03392000 0.16862700<br>N 0.24779200 0.96911300 0.00132900<br>C -0.80130900 -2.56956500 0.42526300<br>C -2.16710900 0.23260700 0.12132400	Zero-point correction= 0.398980 (Hartree/Particle)<br>Thermal correction to Energy= 0.424862<br>Thermal correction to Enthalpy= 0.425806<br>Thermal correction to Gibbs Free Energy= 0.340554<br>Sum of electronic and zero-point Energies= -1353.971883<br>Sum of electronic and thermal Energies= -1353.946001<br>Sum of electronic and thermal Enthalpies= -1353.945057<br>Sum of electronic and thermal Free Energies= -1354.030309

C	5.00255700	0.53113700	-0.48146100	
C	6.07652300	-0.31546100	-0.47046400	
C	5.89942000	-1.70533800	-0.24322000	
C	4.65208100	-2.22184800	-0.02852700	
C	-2.72350800	0.92272400	-0.95596200	
C	-4.09643100	1.12397800	-1.01778100	
C	-4.92018100	0.64576600	-0.00241200	
C	-4.36733900	-0.04142900	1.07450400	
C	-2.99510200	-0.25076900	1.13517400	
C	0.02483600	2.36684200	0.06540100	
C	-1.01261900	2.89357100	0.82898700	
C	-1.30875800	4.25140400	0.76067000	
C	-0.57609700	5.10583800	-0.06887700	
C	0.50207200	4.60423800	-0.77004200	
C	0.89254200	3.24831000	-0.63224600	
O	-0.42403400	-3.46639600	1.12849500	
O	-1.89936300	-2.63226500	-0.34303300	
C	-2.73045700	-3.78822000	-0.16950900	
C	-3.96231100	-3.56957700	-1.01913000	
H	5.10614800	1.59746300	-0.64253400	
H	7.07424500	0.07414100	-0.63351100	
H	6.76404000	-2.35813300	-0.23893200	
H	4.48611200	-3.27745500	0.14918500	
H	-2.07919800	1.29556800	-1.74448100	
H	-4.52418900	1.65444300	-1.85991400	
H	-5.99027800	0.80875600	-0.05046400	
H	-5.00431600	-0.41356200	1.86810300	
H	-2.55695100	-0.78493700	1.97178800	
H	-1.60601400	2.25073800	1.46332900	
H	-2.12763600	4.64085400	1.35329100	
H	-0.84322400	6.15215900	-0.14748900	
H	1.11486800	5.25047400	-1.38582200	
H	1.64156200	2.83294600	-1.28915000	
H	-2.16963400	-4.67672300	-0.46676100	
H	-2.97444400	-3.88605400	0.89149200	
H	-4.65034600	-4.40892700	-0.90382700	
H	-4.47068700	-2.65174100	-0.71677300	
H	-3.69097800	-3.48380000	-2.07221000	
O	2.29249100	3.59404600	0.73554100	
H	1.81961100	4.24333600	1.27916600	
**Name**				**TS-C23-OH-FHT-G**
Cartesian Coordinates				Energy
C	1.21982800	-1.15071100	0.09504700	Zero-point correction= 0.394437 (Hartree/Particle)
C	1.40540000	0.26981200	-0.02532400	Thermal correction to Energy= 0.420284
N	2.55028200	0.87612200	-0.09720500	Thermal correction to Enthalpy= 0.421228
C	3.61830600	0.03265800	-0.05901500	Thermal correction to Gibbs Free Energy= 0.335164
C	3.45584500	-1.38490400	0.03771900	Sum of electronic and zero-point Energies= -1353.986009
N	2.23551000	-1.97541100	0.11351600	Sum of electronic and thermal Energies= -1353.960163
C	-0.20457800	-1.37304800	0.13681400	Sum of electronic and thermal Enthalpies= -1353.959218
C	-0.80829600	-0.13527400	0.06121000	Sum of electronic and thermal Free Energies= -1354.045283
N	0.14958600	0.86247100	-0.03642500	
C	-0.85164500	-2.68924200	0.15952000	
C	-2.24479600	0.19694300	0.07896800	
C	4.92650300	0.57722300	-0.12499900	

C 6.01774500 -0.24626100 -0.09916300<br>C 5.85960200 -1.65379900 -0.00720700<br>C 4.61275800 -2.20908400 0.05977600<br>C -2.79980900 0.98820000 -0.92706900<br>C -4.15643000 1.28805100 -0.90652500<br>C -4.96149400 0.81284800 0.12398000<br>C -4.40829400 0.03073600 1.13440900<br>C -3.05512200 -0.28009400 1.11049200<br>C -0.06708500 2.27342300 0.00288000<br>C -0.83421800 2.83534200 1.01883400<br>C -1.04529000 4.20841500 1.03376200<br>C -0.47770800 5.01529300 0.05254000<br>C 0.30383200 4.44586400 -0.94758100<br>C 0.50879000 3.07170000 -0.97982600<br>O -0.34961300 -3.69628700 0.58938700<br>O -2.08681700 -2.65646800 -0.37863500<br>C -2.86001900 -3.83754500 -0.28587400<br>C -4.19861300 -3.58098700 -0.92480300<br>H 5.01986000 1.65429100 -0.19409300<br>H 7.01452000 0.17538800 -0.14883300<br>H 6.73744900 -2.28826600 0.01113900<br>H 4.45977100 -3.27893300 0.13305800<br>H -2.16990800 1.36185300 -1.72649500<br>H -4.58397300 1.89540200 -1.69496900<br>H -6.01773100 1.05373700 0.14164500<br>H -5.03097700 -0.33593700 1.94177200<br>H -2.61728900 -0.88997700 1.89313500<br>H -1.26454000 2.19944900 1.78302800<br>H -1.64777000 4.64761900 1.81943100<br>H -0.63986300 6.08615800 0.07065700<br>H 0.75340600 5.07099700 -1.70938100<br>H 1.11614900 2.61415900 -1.75036500<br>H -2.30749600 -4.69378500 -0.87118800<br>H -2.90515600 -4.18154200 0.75035100<br>H -4.81681700 -4.47725500 -0.86558800<br>H -4.70803800 -2.76049500 -0.41179000<br>H -4.07209200 -3.30812600 -1.97305800<br>O -1.38089100 -5.61956000 -1.34380300<br>H -0.67996000 -5.37573200 -0.71270500	
**Name**	**TS-C23-OH-FHT-P**
Cartesian Coordinates	Energy
C 1.22063200 -1.15159100 0.07963500<br>C 1.40718900 0.27163700 -0.03610500<br>N 2.55386000 0.87420400 -0.09961900<br>C 3.62437500 0.03186100 -0.06098800<br>C 3.46232900 -1.38577600 0.02961800<br>N 2.23839300 -1.97428100 0.09951100<br>C -0.20247100 -1.37093000 0.11089300<br>C -0.80394300 -0.12935300 0.03529300<br>N 0.15483700 0.86349600 -0.05381400<br>C -0.86336800 -2.67706800 0.15114200<br>C -2.23956800 0.20117800 0.06648200<br>C 4.93192000 0.57806200 -0.12093900<br>C 6.02469800 -0.24516700 -0.09516100	Zero-point correction= 0.394147 (Hartree/Particle)<br>Thermal correction to Energy= 0.419994<br>Thermal correction to Enthalpy= 0.420938<br>Thermal correction to Gibbs Free Energy= 0.335094<br>Sum of electronic and zero-point Energies= -1354.021621<br>Sum of electronic and thermal Energies= -1353.995774<br>Sum of electronic and thermal Enthalpies= -1353.994830<br>Sum of electronic and thermal Free Energies= -1354.080674

C	5.86685300	-1.65270500	-0.00899700	
C	4.61907300	-2.20945200	0.05222200	
C	-2.80192500	1.01642600	-0.91765100	
C	-4.16016900	1.31001600	-0.88064500	
C	-4.95737200	0.80498400	0.14285600	
C	-4.39624100	-0.00255500	1.12937200	
C	-3.04174000	-0.30859000	1.09001300	
C	-0.06378600	2.27689200	-0.00124900	
C	-0.75240400	2.83019300	1.07345500	
C	-0.96966900	4.20286300	1.10589600	
C	-0.48671800	5.01254800	0.08141200	
C	0.21457100	4.44880400	-0.98060400	
C	0.42493300	3.07506200	-1.02973400	
O	-0.40191000	-3.66592500	0.67159900	
O	-2.04946100	-2.66905700	-0.47954200	
C	-2.88298000	-3.80510700	-0.31852900	
C	-4.16753000	-3.56876400	-1.06339700	
H	5.02884600	1.65572700	-0.18695000	
H	7.02171700	0.17709900	-0.14104300	
H	6.74523600	-2.28710900	0.00909500	
H	4.47252800	-3.28131100	0.12058600	
H	-2.18078800	1.40903800	-1.71517400	
H	-4.59499900	1.93546300	-1.65132100	
H	-6.01467800	1.04179500	0.17297000	
H	-5.01314500	-0.39265300	1.93055000	
H	-2.59839000	-0.93362300	1.85835000	
H	-1.11555100	2.18963300	1.86902400	
H	-1.51060200	4.63933000	1.93709100	
H	-0.65449400	6.08273400	0.11247900	
H	0.59420000	5.07678000	-1.77786600	
H	0.95952800	2.62103800	-1.85541000	
H	-2.34051800	-4.73152000	-0.77330100	
H	-3.01182700	-4.03153100	0.74292500	
H	-4.81998200	-4.43644200	-0.95487600	
H	-4.67838000	-2.69009600	-0.65899300	
H	-3.97338300	-3.40342200	-2.12446000	
O	-1.38108900	-5.74897000	-1.08464400	
H	-0.72382400	-5.40843500	-0.45030900	
**Name**				**TS-C24-OH-FHT-G**
Cartesian Coordinates				Energy
C	1.26930000	-1.11057000	0.11587900	Zero-point correction= 0.392923 (Hartree/Particle)
C	1.43588900	0.31427500	-0.00195500	Thermal correction to Energy= 0.418399
N	2.57591100	0.93878200	-0.07077200	Thermal correction to Enthalpy= 0.419343
C	3.65529900	0.10845900	-0.03206700	Thermal correction to Gibbs Free Energy= 0.335743
C	3.51188400	-1.31387900	0.06425400	Sum of electronic and zero-point Energies= -1353.976961
N	2.29945500	-1.92319600	0.13718900	Sum of electronic and thermal Energies= -1353.951485
C	-0.15172400	-1.35385700	0.15593700	Sum of electronic and thermal Enthalpies= -1353.950541
C	-0.77243500	-0.12316600	0.08012900	Sum of electronic and thermal Free Energies= -1354.034140
N	0.17309600	0.89008800	-0.01537300	
C	-0.78471800	-2.68305700	0.16477600	
C	-2.21467400	0.19166100	0.09425800	
C	4.95783800	0.67174800	-0.09585700	
C	6.06336400	-0.13811700	-0.06805700	
C	5.92426600	-1.54999300	0.02382900	

C	4.68166900	-2.12315800	0.08846600	
C	-2.78575500	0.94526200	-0.93455100	
C	-4.14889000	1.22849600	-0.91759800	
C	-4.94504600	0.77478500	0.13306600	
C	-4.37587500	0.02973100	1.16657500	
C	-3.01607100	-0.26507700	1.14520000	
C	-0.06373500	2.29709100	0.01556700	
C	-0.83839900	2.85611400	1.03131800	
C	-1.06885100	4.22900700	1.03892100	
C	-0.51206000	5.04015700	0.05094700	
C	0.27656400	4.47410300	-0.94964800	
C	0.49969700	3.09975200	-0.97478900	
O	-0.26871200	-3.69444600	0.57658300	
O	-2.02524800	-2.64925100	-0.36333200	
C	-2.74344500	-3.88102800	-0.35368600	
C	-4.11087000	-3.59118400	-0.90964200	
H	5.03679100	1.75252700	-0.16491500	
H	7.05642700	0.29872600	-0.11621700	
H	6.81264800	-2.17401400	0.04391300	
H	4.54273600	-3.19744300	0.16170900	
H	-2.16345600	1.29911000	-1.75217000	
H	-4.58848400	1.80399000	-1.72638500	
H	-6.00686800	1.00253200	0.14786500	
H	-4.99132900	-0.32125000	1.98991200	
H	-2.56593400	-0.84893500	1.94422100	
H	-1.26014400	2.21737900	1.80153200	
H	-1.67861600	4.66474300	1.82442600	
H	-0.68920200	6.11129100	0.06339200	
H	0.71722600	5.10188600	-1.71804600	
H	1.11090100	2.64515700	-1.74759100	
H	-2.20297400	-4.62240100	-0.95083100	
H	-2.80059100	-4.25807800	0.67594200	
H	-4.75786800	-4.47124400	-0.91614100	
H	-4.59646500	-2.73292300	-0.43763300	
H	-3.96054900	-3.25370900	-2.05413200	
O	-3.76537500	-3.11318500	-3.37358300	
H	-4.34066400	-3.83178400	-3.70222100	
**Name**				**TS-C25-OH-RAF-G**
Cartesian Coordinates				Energy
C	1.26996200	-1.15408000	-0.21463800	Zero-point correction= 0.398673 (Hartree/Particle)
C	1.39957400	0.27592300	-0.14933400	Thermal correction to Energy= 0.424494
N	2.51828100	0.92456400	-0.02692300	Thermal correction to Enthalpy= 0.425438
C	3.61388400	0.11821900	0.01659100	Thermal correction to Gibbs Free Energy= 0.341286
C	3.50723700	-1.30674300	-0.05537300	Sum of electronic and zero-point Energies= -1353.974072
N	2.31337500	-1.94272600	-0.16397500	Sum of electronic and thermal Energies= -1353.948251
C	-0.13864200	-1.43865900	-0.30125400	Sum of electronic and thermal Enthalpies= -1353.947307
C	-0.79144100	-0.23504700	-0.30648500	Sum of electronic and thermal Free Energies= -1354.031458
N	0.12639500	0.81794700	-0.20956500	
C	-0.73024200	-2.77863300	-0.50991400	
C	-2.23866300	0.08057200	-0.46870100	
C	4.89759800	0.71014700	0.13937000	
C	6.01734800	-0.07304700	0.18525000	
C	5.91459200	-1.48704700	0.11235600	
C	4.69304700	-2.08812800	-0.00404300	

C	-2.59937900	1.16817200	-1.32173500	
C	-3.84962800	1.74612700	-1.23512300	
C	-4.74495900	1.33170000	-0.24671600	
C	-4.38006900	0.33323400	0.66145500	
C	-3.14278900	-0.26919200	0.57822400	
C	-0.15711300	2.19468900	0.02792200	
C	-1.02890900	2.56272400	1.04943900	
C	-1.31592100	3.90663000	1.25368500	
C	-0.71635200	4.87892000	0.45945900	
C	0.17491900	4.50373700	-0.54142800	
C	0.45458700	3.16107000	-0.76480400	
O	-0.28481300	-3.60849200	-1.25151900	
O	-1.82302100	-2.95603700	0.24842000	
C	-2.64965300	-4.07244500	-0.11096200	
C	-3.99110600	-3.85965600	0.55423400	
H	4.94891200	1.79085200	0.19504200	
H	6.99441300	0.38578600	0.27883000	
H	6.81428000	-2.08929700	0.14989700	
H	4.58150800	-3.16403400	-0.06276600	
H	-1.88145500	1.50906300	-2.05942200	
H	-4.12295400	2.54114100	-1.91788900	
H	-5.71940100	1.79939600	-0.17220000	
H	-5.06900000	0.03649500	1.44321600	
H	-2.83993100	-1.03777500	1.27704900	
H	-1.48114000	1.79912600	1.67169000	
H	-2.00170900	4.19273500	2.04187000	
H	-0.93672600	5.92652200	0.62498500	
H	0.65187200	5.25798700	-1.15548800	
H	1.14481600	2.85356100	-1.54015600	
H	-2.73827600	-4.09276400	-1.19856500	
H	-2.16183000	-4.99403800	0.21433800	
H	-4.65791500	-4.69352800	0.32726200	
H	-3.88066700	-3.79247400	1.63863800	
H	-4.44204900	-2.93762600	0.18218200	
O	-2.72925800	-1.36088900	-1.71840000	
H	-3.02633100	-0.84694100	-2.48361500	
**Name**				**TS-C26-OH-RAF-G**
Cartesian Coordinates				Energy
C	1.24167800	-1.07685200	0.11942100	Zero-point correction= 0.399394 (Hartree/Particle)
C	1.42376000	0.34425800	-0.01298300	Thermal correction to Energy= 0.425044
N	2.56865400	0.94956900	-0.09971900	Thermal correction to Enthalpy= 0.425989
C	3.63653900	0.10605200	-0.06867300	Thermal correction to Gibbs Free Energy= 0.341661
C	3.47619800	-1.31159200	0.03681800	Sum of electronic and zero-point Energies= -1353.983195
N	2.25739200	-1.90174800	0.12959500	Sum of electronic and thermal Energies= -1353.957544
C	-0.17977200	-1.30201300	0.17941600	Sum of electronic and thermal Enthalpies= -1353.956600
C	-0.78455000	-0.06729700	0.10374300	Sum of electronic and thermal Free Energies= -1354.040927
N	0.16871800	0.93466800	-0.01358600	
C	-0.83898000	-2.61944400	0.22292900	
C	-2.22197300	0.23714600	0.12375100	
C	4.94402600	0.65025100	-0.15243000	
C	6.03579600	-0.17262400	-0.13474800	
C	5.87956400	-1.57989500	-0.03362800	
C	4.63388900	-2.13517400	0.05037900	
C	-2.79543500	1.00806400	-0.92282900	

C	-4.17693900	1.31213100	-0.85864000	
C	-4.96919400	0.77017400	0.13212200	
C	-4.40314000	-0.05040400	1.11440600	
C	-3.03707200	-0.31356200	1.10626100	
C	-0.06117300	2.34163000	0.02741700	
C	-0.84913200	2.89106600	1.03488900	
C	-1.08471900	4.26058400	1.04612800	
C	-0.51939100	5.07663500	0.07144400	
C	0.28382000	4.51994900	-0.91898400	
C	0.51203300	3.14957500	-0.94953200	
O	-0.42020500	-3.58425300	0.79884800	
O	-1.99321300	-2.62394900	-0.48081300	
C	-2.80076400	-3.80870400	-0.37697400	
C	-4.07071700	-3.53953500	-1.15345700	
H	5.03625500	1.72694100	-0.22845300	
H	7.03170700	0.24925600	-0.19814100	
H	6.75794300	-2.21378300	-0.02159400	
H	4.48204800	-3.20464000	0.13147000	
H	-2.15098400	1.59147400	-1.56619500	
H	-4.60625900	1.94261800	-1.62671700	
H	-6.03078700	0.98449200	0.15516200	
H	-5.02608900	-0.47211800	1.89405900	
H	-2.59434200	-0.93532500	1.87690400	
H	-1.27280400	2.24927000	1.79798100	
H	-1.70296000	4.68972400	1.82512500	
H	-0.70025500	6.14451800	0.08673600	
H	0.73023700	5.15227700	-1.67668000	
H	1.13125000	2.70122900	-1.71577700	
H	-2.23763500	-4.65438400	-0.77557800	
H	-2.99596700	-4.00212900	0.68055700	
H	-4.74126100	-4.39720900	-1.07883500	
H	-4.58098400	-2.65849200	-0.75763100	
H	-3.85106500	-3.36320900	-2.20793900	
O	-2.84182200	-0.37720700	-2.33722100	
H	-2.64137900	-1.16235200	-1.79828900	
**Name**				**TS-C27-OH-RAF-G**
Cartesian Coordinates				Energy
C	1.23914300	-1.07877600	0.10384400	Zero-point correction= 0.399082 (Hartree/Particle)
C	1.42682400	0.34437200	0.00486700	Thermal correction to Energy= 0.424868
N	2.57288200	0.94935000	-0.06364700	Thermal correction to Enthalpy= 0.425812
C	3.63934000	0.10337800	-0.04376000	Thermal correction to Gibbs Free Energy= 0.340969
C	3.47472000	-1.31517800	0.03142900	Sum of electronic and zero-point Energies= -1353.979276
N	2.25385800	-1.90474300	0.10369900	Sum of electronic and thermal Energies= -1353.953491
C	-0.18545900	-1.30103200	0.14885200	Sum of electronic and thermal Enthalpies= -1353.952547
C	-0.78312800	-0.06280300	0.09750000	Sum of electronic and thermal Free Energies= -1354.037390
N	0.17279300	0.93888700	0.00990000	
C	-0.84236400	-2.62016500	0.16898900	
C	-2.22326800	0.24801100	0.10382300	
C	4.94837300	0.64632700	-0.10774100	
C	6.03818000	-0.17944700	-0.09998600	
C	5.87786100	-1.58797000	-0.02894100	
C	4.63021500	-2.14176400	0.03542500	
C	-2.78161400	0.99625700	-0.92140500	
C	-4.18561100	1.14773600	-1.00297500	

C -4.98429000 0.69672600 0.07690500<br>C -4.41271300 -0.01277800 1.11619200<br>C -3.03926400 -0.25729200 1.12363700<br>C -0.05210700 2.34709600 0.05330600<br>C -0.83844200 2.89881600 1.06059200<br>C -1.06095200 4.27015700 1.07905500<br>C -0.48556600 5.08632900 0.11019500<br>C 0.31469600 4.52759100 -0.88114900<br>C 0.53079100 3.15529200 -0.91755700<br>O -0.36092500 -3.61357600 0.64069500<br>O -2.05863800 -2.59508400 -0.41546500<br>C -2.81008900 -3.81668400 -0.33341500<br>C -4.15296300 -3.55899000 -0.97864400<br>H 5.04358300 1.72417100 -0.16048100<br>H 7.03548800 0.24119000 -0.14783600<br>H 6.75461000 -2.22421800 -0.02398800<br>H 4.47512300 -3.21225400 0.09371100<br>H -2.15739700 1.39563500 -1.71214600<br>H -4.60030600 1.84736200 -1.71486400<br>H -6.05202900 0.87386700 0.05064900<br>H -5.02866200 -0.38596200 1.92532400<br>H -2.58990100 -0.83653000 1.92271200<br>H -1.27179600 2.25645900 1.81766700<br>H -1.67706800 4.70095900 1.85882500<br>H -0.65619000 6.15579800 0.13116200<br>H 0.76990400 5.15985600 -1.63365400<br>H 1.15217800 2.70665000 -1.68199900<br>H -2.25306300 -4.60831100 -0.83807200<br>H -2.90745300 -4.09641000 0.71831400<br>H -4.77819700 -4.45040000 -0.90429900<br>H -4.66865100 -2.73328000 -0.48259100<br>H -4.03663200 -3.31496400 -2.03662000<br>O -4.51016500 -0.29793600 -2.30970000<br>H -3.93171500 -0.97803200 -1.92687500	
**Name**	**TS-C28-OH-RAF-G**
Cartesian Coordinates	Energy
C 1.29436000 -1.14914300 0.03619500<br>C 1.43289500 0.28144000 -0.01953800<br>N 2.55546500 0.93229900 -0.04302800<br>C 3.65265800 0.12706200 -0.02317300<br>C 3.53934400 -1.29816200 0.01186600<br>N 2.34100700 -1.93505500 0.04186100<br>C -0.12361900 -1.42585600 0.05317700<br>C -0.76599600 -0.20728100 0.01309100<br>N 0.15878400 0.82901900 -0.02894900<br>C -0.72488300 -2.76947800 0.01558400<br>C -2.20156100 0.12510100 -0.01736800<br>C 4.94142000 0.71963100 -0.04368800<br>C 6.06073700 -0.06554800 -0.03268100<br>C 5.95178100 -1.48058800 -0.00109100<br>C 4.72477700 -2.08154400 0.02097300<br>C -2.70290000 0.90247200 -1.06928700<br>C -4.02396700 1.30555900 -1.06638300<br>C -4.85299000 1.01540200 0.04358300	Zero-point correction= 0.398593 (Hartree/Particle)<br>Thermal correction to Energy= 0.424700<br>Thermal correction to Enthalpy= 0.425644<br>Thermal correction to Gibbs Free Energy= 0.338513<br>Sum of electronic and zero-point Energies= -1353.979878<br>Sum of electronic and thermal Energies= -1353.953771<br>Sum of electronic and thermal Enthalpies= -1353.952827<br>Sum of electronic and thermal Free Energies= -1354.039958

C -4.35797000 0.15402200 1.05373000<br>C -3.04746200 -0.27099900 1.02768400<br>C -0.12275400 2.22289600 0.09621200<br>C -0.92000300 2.68095400 1.14293100<br>C -1.21404200 4.03612200 1.23603800<br>C -0.69754000 4.92999100 0.30182700<br>C 0.12030300 4.46599400 -0.72298300<br>C 0.40797900 3.11000900 -0.83303800<br>O -0.12719400 -3.79183700 0.21417000<br>O -2.03786200 -2.72420600 -0.27781900<br>C -2.71952000 -3.98564100 -0.31372700<br>C -4.17478300 -3.68921400 -0.60002800<br>H 4.99730200 1.80127900 -0.06648600<br>H 7.04248700 0.39251400 -0.04746500<br>H 6.85148300 -2.08389900 0.00719100<br>H 4.60803500 -3.15813900 0.04779300<br>H -2.04603600 1.18386400 -1.88444400<br>H -4.41786100 1.90143700 -1.88010200<br>H -5.91572400 1.20090300 -0.02289700<br>H -5.00747000 -0.12258500 1.87467900<br>H -2.65302200 -0.89756700 1.81896100<br>H -1.30717000 1.97946100 1.87293100<br>H -1.83751300 4.39385700 2.04727500<br>H -0.92613400 5.98590000 0.37866300<br>H 0.53290400 5.15956000 -1.44545300<br>H 1.04124700 2.73412200 -1.62673300<br>H -2.26505100 -4.60961800 -1.08623800<br>H -2.58143200 -4.48968200 0.64534100<br>H -4.74382700 -4.61952900 -0.64253900<br>H -4.59756900 -3.05616500 0.18192300<br>H -4.27939000 -3.17224800 -1.55517500<br>O -4.69238000 2.77230900 0.98420400<br>H -3.75360100 2.95248300 0.81270000	
**Name**	**TS-C29-OH-RAF-G**
Cartesian Coordinates	Energy
C 1.28468200 -1.12469600 -0.04302000<br>C 1.42923100 0.30615700 -0.02877200<br>N 2.55277000 0.95476600 0.00338300<br>C 3.64721600 0.14547300 0.00986700<br>C 3.52931500 -1.27958600 -0.01227100<br>N 2.32894100 -1.91364600 -0.03534800<br>C -0.13554500 -1.39585400 -0.05777500<br>C -0.77240200 -0.17336800 -0.05259300<br>N 0.15754900 0.85682100 -0.03525800<br>C -0.74045200 -2.73120200 -0.18543900<br>C -2.20548300 0.18019400 -0.14461000<br>C 4.93779100 0.73380500 0.04016800<br>C 6.05455000 -0.05508100 0.04613200<br>C 5.94107600 -1.46986400 0.02307700<br>C 4.71216700 -2.06679700 -0.00497800<br>C -2.64976300 0.89070400 -1.24904900<br>C -3.98748600 1.34602700 -1.31944500<br>C -4.88734900 0.96122800 -0.29595600<br>C -4.43686200 0.24506200 0.79757400	Zero-point correction= 0.398686 (Hartree/Particle)<br>Thermal correction to Energy= 0.424670<br>Thermal correction to Enthalpy= 0.425614<br>Thermal correction to Gibbs Free Energy= 0.339845<br>Sum of electronic and zero-point Energies= -1353.980111<br>Sum of electronic and thermal Energies= -1353.954127<br>Sum of electronic and thermal Enthalpies= -1353.953183<br>Sum of electronic and thermal Free Energies= -1354.038953

C	-3.10059200	-0.14710500	0.88026600	
C	-0.12306400	2.24677800	0.13039800	
C	-0.84534800	2.67701900	1.24042400	
C	-1.13397500	4.02945800	1.38565200	
C	-0.69075000	4.94409500	0.43401700	
C	0.04981500	4.50604200	-0.65871500	
C	0.33549700	3.15475000	-0.81609200	
O	-0.12801700	-3.74618600	-0.37622300	
O	-2.08191700	-2.68885800	-0.06953500	
C	-2.77094200	-3.93382800	-0.24489600	
C	-4.24559500	-3.64311800	-0.07442000	
H	4.99687600	1.81536900	0.05887000	
H	7.03761000	0.39983800	0.06933600	
H	6.83870200	-2.07631900	0.02829900	
H	4.59199800	-3.14321300	-0.02293900	
H	-1.96157400	1.14507800	-2.04692800	
H	-4.37549200	1.69958300	-2.26422000	
H	-5.92299100	1.26941000	-0.36446800	
H	-5.12301700	-0.02112300	1.59248700	
H	-2.74551100	-0.71821500	1.72960900	
H	-1.17630000	1.95443200	1.97725300	
H	-1.70167900	4.36756100	2.24416400	
H	-0.92152800	5.99624400	0.54613900	
H	0.39829200	5.21613800	-1.39837100	
H	0.90817800	2.79688000	-1.66277800	
H	-2.54088500	-4.32715900	-1.23748700	
H	-2.40210700	-4.65060400	0.49173600	
H	-4.82646300	-4.55505100	-0.22281000	
H	-4.44815500	-3.26179700	0.92810000	
H	-4.57375600	-2.89568000	-0.79901500	
O	-3.68266300	3.25282500	-0.88301200	
H	-3.03655300	3.16741100	-0.16307000	
**Name**				**TS-C30-OH-RAF-G**
Cartesian Coordinates				Energy
C	1.25028400	-1.07829200	0.12100400	Zero-point correction= 0.399395 (Hartree/Particle)
C	1.43350500	0.34272200	-0.01086900	Thermal correction to Energy= 0.425045
N	2.57894800	0.94736700	-0.09493800	Thermal correction to Enthalpy= 0.425990
C	3.64626300	0.10322000	-0.06150500	Thermal correction to Gibbs Free Energy= 0.341660
C	3.48485200	-1.31433400	0.04352700	Sum of electronic and zero-point Energies= -1353.983194
N	2.26549000	-1.90378200	0.13345800	Sum of electronic and thermal Energies= -1353.957544
C	-0.17143600	-1.30261900	0.17770500	Sum of electronic and thermal Enthalpies= -1353.956599
C	-0.77530900	-0.06753800	0.10075000	Sum of electronic and thermal Free Energies= -1354.040929
N	0.17881700	0.93387000	-0.01430500	
C	-0.83152500	-2.61966200	0.21958300	
C	-2.21259500	0.23775500	0.11748900	
C	4.95425600	0.64666500	-0.14222400	
C	6.04550300	-0.17684600	-0.12208500	
C	5.88821800	-1.58403300	-0.02142100	
C	4.64203200	-2.13859100	0.05969100	
C	-2.78321000	1.00906100	-0.93036000	
C	-4.16468800	1.31390100	-0.86932600	
C	-4.95951800	0.77234700	0.11959400	
C	-4.39618400	-0.04858500	1.10314200	
C	-3.03025100	-0.31252100	1.09811400	

C -0.05033700 2.34096500 0.02630600<br>C -0.84031200 2.89076000 1.03200000<br>C -1.07511000 4.26041800 1.04284200<br>C -0.50703400 5.07623700 0.06956300<br>C 0.29814100 4.51918000 -0.91905800<br>C 0.52561300 3.14867400 -0.94922400<br>O -0.41462500 -3.58478200 0.79633800<br>O -1.98417000 -2.62340400 -0.48676400<br>C -2.79267600 -3.80767500 -0.38484200<br>C -4.06076000 -3.53762400 -1.16406900<br>H 5.04728700 1.72330600 -0.21796000<br>H 7.04180300 0.24445700 -0.18315300<br>H 6.76619800 -2.21843300 -0.00742500<br>H 4.48938500 -3.20797400 0.14036300<br>H -2.13694600 1.59215100 -1.57219800<br>H -4.59189000 1.94465900 -1.63836200<br>H -6.02104200 0.98726000 0.14020300<br>H -5.02115700 -0.46997600 1.88134800<br>H -2.58963900 -0.93457000 1.86973900<br>H -1.26614600 2.24913100 1.79402800<br>H -1.69490300 4.68984400 1.82044600<br>H -0.68729500 6.14422600 0.08455100<br>H 0.74669400 5.15132500 -1.67564500<br>H 1.14634500 2.70004200 -1.71407500<br>H -2.22919400 -4.65365100 -0.78231800<br>H -2.99030900 -4.00111600 0.67223400<br>H -4.73200500 -4.39488500 -1.09101600<br>H -4.57133700 -2.65630400 -0.76925800<br>H -3.83869600 -3.36130900 -2.21804800<br>O -2.82716100 -0.37607500 -2.34496000<br>H -2.62838500 -1.16137400 -1.80563500	
**Name**	**TS-N10-OH-RAF-G**
Cartesian Coordinates	Energy
C 1.30435600 -1.06884100 -0.08256700<br>C 1.46537300 0.34609900 -0.07160300<br>N 2.59244700 0.99794800 -0.04317000<br>C 3.68842900 0.18608400 -0.03436300<br>C 3.59235500 -1.23464200 -0.07554500<br>N 2.37065000 -1.86375800 -0.10298600<br>C -0.10430300 -1.34100600 -0.08259600<br>C -0.74512100 -0.11154400 -0.06743000<br>N 0.19190700 0.90493400 -0.05843900<br>C -0.69305300 -2.68160800 -0.18054300<br>C -2.18441800 0.21744300 -0.09230300<br>C 4.97639800 0.77552000 0.02030600<br>C 6.09965800 -0.00690200 0.01666900<br>C 5.99407700 -1.41720600 -0.04470900<br>C 4.76521000 -2.02039900 -0.09234000<br>C -2.68978300 1.02563400 -1.11133800<br>C -4.03818900 1.36129400 -1.13092600<br>C -4.88438500 0.90517900 -0.12557600<br>C -4.38060900 0.10636000 0.89764000<br>C -3.03634300 -0.24078300 0.91275800<br>C -0.05801600 2.30597100 0.06640300	Zero-point correction= 0.400104 (Hartree/Particle)<br>Thermal correction to Energy= 0.425531<br>Thermal correction to Enthalpy= 0.426475<br>Thermal correction to Gibbs Free Energy= 0.342264<br>Sum of electronic and zero-point Energies= -1353.977514<br>Sum of electronic and thermal Energies= -1353.952087<br>Sum of electronic and thermal Enthalpies= -1353.951143<br>Sum of electronic and thermal Free Energies= -1354.035355

C	-0.86409600	2.78329200	1.09516300	
C	-1.10870000	4.14735500	1.19257800	
C	-0.53625200	5.02853500	0.28054300	
C	0.28402300	4.54273300	-0.73269500	
C	0.52361900	3.17865900	-0.84702900	
O	-0.05374200	-3.70761000	-0.15417100	
O	-2.02431100	-2.66217600	-0.30499100	
C	-2.67348700	-3.93704100	-0.42903100	
C	-4.15865600	-3.66296100	-0.50528000	
H	5.02865900	1.85671500	0.06001600	
H	7.07921100	0.45440600	0.05553200	
H	6.89291900	-2.02136600	-0.05868800	
H	4.64744900	-3.09359900	-0.16563600	
H	-2.02503100	1.38826800	-1.88743900	
H	-4.42584700	1.98247700	-1.92917900	
H	-5.93422400	1.17305800	-0.13736300	
H	-5.03664600	-0.24685800	1.68444800	
H	-2.63881100	-0.86897500	1.70122900	
H	-1.29882400	2.08959500	1.80442200	
H	-1.74141400	4.52119500	1.98824600	
H	-0.72495000	6.09205400	0.36272900	
H	0.73787000	5.22588200	-1.44022200	
H	1.16267700	2.78536600	-1.62734700	
H	-2.29792600	-4.43446300	-1.32564900	
H	-2.40857600	-4.55341000	0.43233500	
H	-4.70437500	-4.60060900	-0.62367700	
H	-4.50486700	-3.16954100	0.40427800	
H	-4.38488700	-3.01550700	-1.35388800	
O	2.37187000	-2.99499100	-1.47163800	
H	1.73818600	-3.61614600	-1.07742100	

References

1. E. Pollak and P. Pechukas, *J. Am. Chem. Soc.*, 1978, **100**, 2984-2991.
2. A. Fernández-Ramos, B. A. Ellingson, R. Meana-Pañeda, J. M. Marques and D. G. Truhlar, *Theor. Chem. Acc.*, 2007, **118**, 813-826.
3. C. Eckart, *Phy. Rev.*, 1930, **35**, 1303.
4. R. A. Marcus, *Annu. Rev. Phys. Chem.*, 1964, **15**, 155-196.
5. R. A. Marcus, *Rev. Mod. Phys.*, 1993, **65**, 599.
6. Y. Lu, A. Wang, P. Shi and H. Zhang, *PloS one*, 2017, **12**, e0169773.
7. Y. Lu, A. Wang, P. Shi, H. Zhang and Z. Li, *PloS one*, 2015, **10**, e0133259.
8. S. F. Nelsen, S. C. Blackstock and Y. Kim, *J. Am. Chem. Soc.*, 1987, **109**, 677-682.
9. S. F. Nelsen, M. N. Weaver, Y. Luo, J. R. Pladziewicz, L. K. Ausman, T. L. Jentzsch and J. J. O'Konek, *J. Phys. Chem. A*, 2006, **110**, 11665-11676.
10. A. Galano and J. R. Alvarez-Idaboy, *J. Comput. Chem.*, 2013, **34**, 2430-2445.
11. F. C. Collins and G. E. Kimball, *J. Colloid Sci.* , 1949, **4**, 425-437.
12. M. Von Smoluchowski, *Z. Phys. Chem*, 1917, **92**, 129-168.
13. D. G. Truhlar, *J. Chem. Educ.*, 1985, **62**, 104.
14. A. Einstein, *Ann. Phys.*, 1905, **17**, 549-560.
15. G. G. Stokes, *Mathematical and Physical Papers*, University Press, Cambridge, 1905.
16. A. Galano and J. Raúl Alvarez-Idaboy, *Int. J. Quantum Chem.*, 2019, **119**, e25665.
17. Q. V. Vo, T. V. Gon, M. V. Bay and A. Mechler, *J. Phys. Chem. B*, 2019, **123**, 10672-10679.
18. Q. V. Vo, M. V. Bay, P. C. Nam, D. T. Quang, M. Flavel, N. T. Hoa and A. Mechler, *J. Org. Chem.*, 2020, **85**, 15514–15520.
19. Y. Okuno, *Chem.: Eur. J.*, 1997, **3**, 212-218.
20. S. Benson, *The foundations of chemical kinetics:* , Malabar, Florida, 1982.
21. C. Iuga, J. R. Alvarez-Idaboy and A. Vivier-Bunge, *J. Phys. Chem. B*, 2011, **115**, 12234-12246.
22. J. R. Alvarez-Idaboy, L. Reyes and N. Mora-Diez, *Org. Biomol. Chem.*, 2007, **5**, 3682-3689.
23. T. H. Le, T. T. Tran and L. K. Huynh, *Chemom. Intell. Lab. Syst.*, 2018, **172**, 10-16.
